# Transgenic Overexpression of the Disordered Prion Protein N1 Fragment in Mice Does Not Protect Against Neurodegenerative Diseases Due to Impaired ER Translocation

**DOI:** 10.1007/s12035-020-01917-2

**Published:** 2020-05-04

**Authors:** Behnam Mohammadi, Luise Linsenmeier, Mohsin Shafiq, Berta Puig, Giovanna Galliciotti, Camilla Giudici, Michael Willem, Thomas Eden, Friedrich Koch-Nolte, Yu-Hsuan Lin, Jörg Tatzelt, Markus Glatzel, Hermann C. Altmeppen

**Affiliations:** 1 Institute of Neuropathology, University Medical Center Hamburg-Eppendorf (UKE), Hamburg, Germany; 2 Department of Neurology, Experimental Research in Stroke and Inflammation (ERSI), University Medical Center Hamburg-Eppendorf, Hamburg, Germany; 3 Biomedical Center (BMC), Faculty of Medicine, Ludwig-Maximilians-University, Munich, Germany; 4 Institute of Immunology, University Medical Center Hamburg-Eppendorf (UKE), Hamburg, Germany; 5 Institute of Biochemistry and Pathobiochemistry, Biochemistry of Neurodegenerative Diseases, Ruhr University Bochum, Bochum, Germany

**Keywords:** Cell-penetrating peptide, Cytosolic prions, Intrinsically disordered domains, Prion disease, Proteasome, Translocation

## Abstract

The structurally disordered N-terminal half of the prion protein (PrP^C^) is constitutively released into the extracellular space by an endogenous proteolytic cleavage event. Once liberated, this N1 fragment acts neuroprotective in ischemic conditions and interferes with toxic peptides associated with neurodegenerative diseases, such as amyloid-beta (Aβ) in Alzheimer’s disease. Since analog protective effects of N1 in prion diseases, such as Creutzfeldt-Jakob disease, have not been studied, and given that the protease releasing N1 has not been identified to date, we have generated and characterized transgenic mice overexpressing N1 (TgN1). Upon intracerebral inoculation of TgN1 mice with prions, no protective effects were observed at the levels of survival, clinical course, neuropathological, or molecular assessment. Likewise, primary neurons of these mice did not show protection against Aβ toxicity. Our biochemical and morphological analyses revealed that this lack of protective effects is seemingly due to an impaired ER translocation of the disordered N1 resulting in its cytosolic retention with an uncleaved signal peptide. Thus, TgN1 mice represent the first animal model to prove the inefficient ER translocation of intrinsically disordered domains (IDD). In contrast to earlier studies, our data challenge roles of cytoplasmic N1 as a cell penetrating peptide or as a potent “anti-prion” agent. Lastly, our study highlights both the importance of structured domains in the nascent chain for proteins to be translocated and aspects to be considered when devising novel N1-based therapeutic approaches against neurodegenerative diseases.

**Electronic supplementary material:**

The online version of this article (10.1007/s12035-020-01917-2) contains supplementary material, which is available to authorized users.

## Introduction

**Fig. 1:**
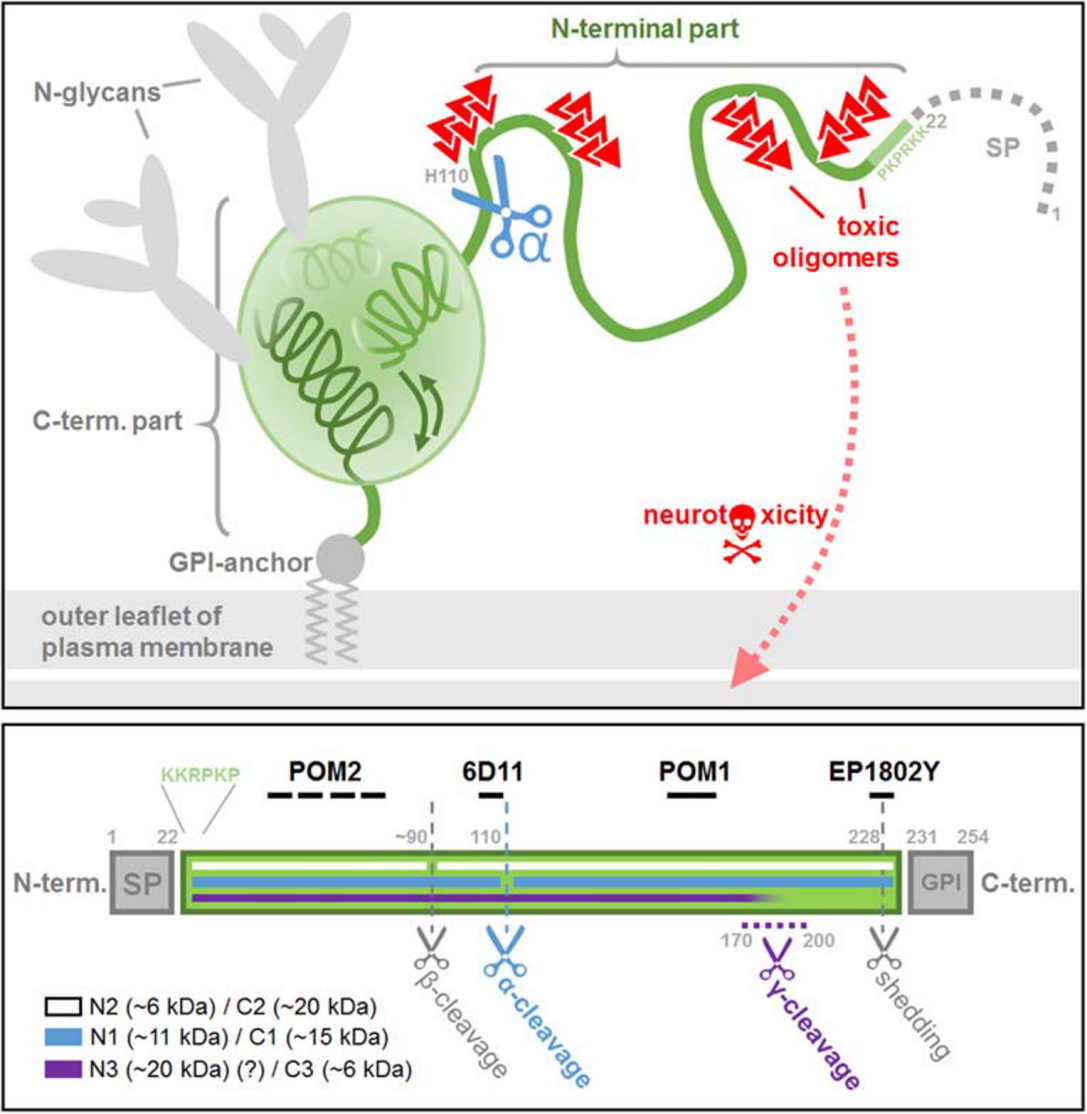
Upper panel: schematic representation of the prion protein. The cellular prion protein (PrP^C^) is composed of two structurally different parts. The rather globular shaped C-terminal half contains three α-helices, a short β-sheet, a disulfide bridge (not shown), up to two N-glycans and a GPI-anchor for attachment to the outer leaflet of the plasma membrane. The N-terminal half is largely unstructured and qualifies as an intrinsically disordered domain (IDD). However, this part contains important binding sites for interaction with various molecules, including toxic protein oligomers and proteopathic seeds (red) found in neurodegenerative diseases. Binding of the latter to membrane-bound PrP^C^ induces neurotoxic signaling events and may lead to pore formation via insertion of the N-terminus into the membrane. A conserved proteolytic cleavage event termed α-cleavage (blue scissors) at H110 separates the two dissimilar halves of PrP^C^ and releases the disordered N1 fragment into the extracellular space, where it exerts neuroprotective effects. Note that the N-terminal ER targeting signal peptide (SP; dotted gray stretch) is not part of mature PrP^C^ under physiological conditions. Lower panel: linear representation of the PrP^C^ sequence accentuating (i) known (proteolytic) cleavage events, (ii) resulting N- and C-terminal fragments (respective expected molecular weights), and (iii) (approximated) position of epitopes described for PrP^C^-directed antibodies (black) used in this study. Note that antibody 6D11 can be instrumental to differentiate between N-terminal fragments resulting from the α- (detection of N1) and β-cleavage (no detection of N2). GPI = position of GPI-anchor signal sequence

The prion protein (PrP^C^), a glycoprotein with high expression levels and biological relevance in the nervous system, is composed of two structurally very dissimilar parts [1–3]. Its globular C-terminal half contains structure-lending elements, such as α-helices, β-sheets, a disulfide bond, up to two N-glycans, and a GPI-anchor for cell surface attachment [4]. In contrast, the N-terminal half of the molecule is highly flexible and lacks structural features [5], thus representing a prototype of so-called *intrinsically disordered domains/proteins* (IDD/IDP, Fig. 1) [6]. Although such IDDs inherently miss folded parts as preformed “binding interfaces”, they are nevertheless able to initiate transient high-affinity interactions with other proteins [7]. In fact, the N-terminal half of PrP^C^ plays key roles for the physiological functions and pathological roles of this protein. As part of the membrane-bound full-length PrP^C^, the N-terminal half is considered as a flexible molecular sensor of the extracellular milieu [8] that may transiently adopt a particular shape upon binding of specific ligands [9]. Several of those interacting ligands have been described, among them divalent cations as well as various proteins/peptides of physiological or pathological relevance [8, 10–13]. Such interactions, for instance, regulate the cellular trafficking and surface homeostasis of PrP^C^ [10, 14–19]. Of note, the N-terminal part is also critical for the initial interaction between PrP^C^ and aggregates (“seeds”) of its pathological isoform (PrP^Sc^) [8, 20–25] and, hence, for the progressive templated misfolding process underlying fatal and transmissible prion diseases in humans (e.g., Creutzfeldt-Jakob disease) and animals (e.g., Scrapie of sheep, BSE in cattle) [26–28]. In these diseases, the N-terminal domain within PrP^C^ also fulfills “toxic effector” functions [3, 29, 30]. It is conceivable that this is due to interactions of this part with the plasma membrane and subsequent pore formation therein [3, 31, 32]. This, in turn, might be linked to the very N-terminal sequence qualifying as a *cell-penetrating peptide* (CPP) [33–35]. Moreover, PrP^C^ at the neuronal surface is a receptor for other toxic oligomeric proteins abundantly produced in other neurodegenerative diseases, such as Alzheimer’s (AD) or Parkinson’s disease (PD). Again, it is the N-terminal part of PrP^C^ that acts as the crucial docking hub for β-sheet-rich oligomeric amyloid-β (Aβ) peptides and hyperphosphorylated tau (pTau) in AD or α-synuclein in PD, thus allowing for high-affinity binding and subsequent neurotoxic signaling via additional PrP^C^ interacting transmembrane proteins (Fig. 1) [9, 36–45].

Interestingly, the N-terminal half can be cleaved off from the protein and is then released into the extracellular space as a soluble N1 fragment in a physiological proteolytic process termed α-cleavage (Fig. 1) [46–48]. This cleavage is evolutionary conserved, occurs constitutively on a considerable fraction of PrP^C^ molecules depending on tissue and cell type, and seems to take place while traversing the secretory pathway or in an endocytic compartment [49]. Biological relevance of this cleavage is supported by the fact that deletions in the α-cleavage region result in severe neurotoxicity in respective transgenic mouse models [50–55]. Fittingly, the α-cleavage has so far mostly been linked with protective effects [56–60]. As a soluble ligand, N1 acts beneficial in several ways as it reduces hypoxia-induced neuronal damage [61] and may be involved in myelin maintenance [62] and intercellular communication (reviewed in [63]). With regard to AD, numerous studies have shown that exogenously administered N1 and N1-like peptides are able to block toxic Aβ oligomers and interfere with their synaptic impairment and neurotoxicity [38, 64–71].

For prion diseases, however, insight into a potential protective role of N1 by similarly neutralizing PrP^Sc^ oligomers is lacking to date. In addition, the responsible protease (termed αPrPase) [72] for the endogenous generation of N1 has not been identified unequivocally to date [73, 74], thus precluding direct pharmacological manipulation. We therefore decided to address this issue in vivo by generating transgenic mice overexpressing N1 and challenging them with prions. Such a mouse model has previously been predicted to be of “crucial importance” to assess the therapeutic potential of N1 in neurodegenerative conditions [75]. While our “protective” strategy eventually failed due to impaired secretion of transgenic N1, our study (i) provides the first proof of the recently described inefficient translocation of IDDs [6] in an animal model. Moreover, it (ii) challenges both, toxic CPP-like and protective antiprion effects of cytosolic N1 with uncleaved signal peptides (N1-SP), (iii) questions a relevant role of cytosolic prions in prion diseases, and (iv) highlights important aspects to be considered when investigating the α-cleavage of PrP^C^ or devising N1-based therapeutic approaches in the future.

## Material and Methods

### Plasmids

To generate the N1-coding plasmid, the construct containing the mouse *Prnp* open reading frame in pcDNA3.1(-)/Zeo expression vector was used to insert a stop codon after H110 of murine PrP using the QuickChange Site-Directed Mutagenesis Kit (Agilent Technologies). For the N1Fc construct, the pFUSE-mIgG1-Fc1 vector (InvivoGen) was purchased and the sequence coding for amino acids (aa) 1–110 of mPrP was subcloned into that preceding the hinge region and the CH2 and CH3 domains of the IgG heavy chain. For generating the N1Nb, the cDNA coding for aa 23–110 of mPrP was genetically fused to the N-terminus of nanobody l-10e via a 5 GS linkage region and subcloned into the pCSE2.5 expression vector [76] (kindly provided by Dr. Thomas Schirrmann, Braunschweig, Germany) containing an IgKappa leader N-terminal of the N1Nb construct and a His/Myc tag at the C-terminus. Primers are listed in Suppl. Table 1B.

### Generation of TgN1 Mice

To generate mice overexpressing the N1 fragment, a stop codon was inserted into the murine *Prnp* sequence (resulting in a stop after H110) in the half-genomic expression vector (mPrPHGC [77]) using the QuickChange II XL Site-Directed Mutagenesis Kit. Before pronuclear injection, the resulting N1mPrPHGC vector was cut with SalI and NotI to remove the pBlue-script sequence. The pronuclear injection into C57BL/6J mice was performed at the Transgenic Mouse Facility (ZMNH, UKE, Hamburg). Positive heterozygous animals were selected by genotyping. All primers are listed in Suppl. Table 1B.

### Prion Inoculations

Intracerebral inoculations of TgN1 mice and WT littermates with prions were performed under deep anesthesia with ketamine and xylazine hydrochloride. In brief, 10–11-week-old TgN1 mice (*n* = 10) and littermate controls (*n* = 9) were inoculated with 30 μL of a 1% homogenate of Rocky Mountain Laboratory (RML) prions (RML 5.0 inoculum, corresponding to 3 × 10^5^LD50) into the forebrain. After inoculation, to minimize suffering of the animals, careful observation and special treatment (such as incubation on a warming plate and administration of wet food) were applied until initial recovery. Mice were then checked daily and observation was intensified upon appearance of first clinical signs of prion disease. All mice were sacrificed and analyzed when they reached fully established prion disease. Additionally, we performed mock inoculations with 30 μL of a 1% brain homogenate from uninfected CD1 mice into age-matched TgN1 (*n* = 5) and littermate controls (*n* = 4). These animals were sacrificed at 240 days post-inoculation (dpi) lacking any clinical signs.

### Primary Neuronal Cultures

#### Monocultures

Primary neurons were prepared from TgN1 and WT littermates at postnatal day 1 (P1). Briefly, after dissecting out the pups’ brains, meninges were removed from both hemispheres. Brain tissues were washed once with pre-cooled dissecting media (DM; 1× HBSS, 1% penicillin-streptomycin, 10 mM HEPES, 0.6% glucose solution). The brains were chopped and tissue pieces were transferred into a 60-mm dish with a total volume of 4.5 mL DM plus 0.5 mL pre-warmed 2.5% Trypsin (Thermo Fisher Scientific), incubated at 37 °C for 15 min under horizontal agitation.

After enzymatic digestion, 100 μL per dish of sterile 1 mg/mL DNase I was added into dishes and gently swirled. The enzymatic activity was then quenched after 1 min with 5 mL of glial growth medium (GGM; DMEM + 0.6% glucose solution, 1% penicillin-streptomycin, 10% FBS); the solution was gently pipetted up and down 2–3 times (on-dish trituration), transferred into a new 15-mL falcon tube, and centrifuged for 5 min at 1000 rpm.

The pellet was resuspended in 5-mL neuronal maintenance medium (NMM; 1% Glutamax with a final concentration of 2 mM, 2% B27 serum supplement, 1% penicillin-streptomycin, in 50-mL neurobasal medium), and cells were dissociated by 15 times pipetting up and down. After filtration through a 70 μm cell strainer, cells were seeded onto PLL-coated dishes (poly-L-lysine hydrobromide, Sigma-Aldrich). The media was changed after 4-h incubation at 37 °C and 5% CO_2_. The next day, cells were treated with 10 μM of the mitotic inhibitor fluorodeoxyuridine (FUdR, Sigma-Aldrich) overnight in order to eliminate non-neuronal cells. On day 5 post-dissection, cells and conditioned media were either harvested for analysis of protein expression or neurons were treated overnight with 5 μM of proteasomal inhibitor (MG132) or left untreated in OptiMEM followed by harvesting.

#### Co-culturing Primary Neurons on an Astrocyte Feeder Layer

For morphological assessment, hippocampal neurons were co-cultured with hippocampal astrocytes following a previously published protocol [78]. Astrocytes were prepared 3 weeks before the day of neuronal dissection. For preparation of the astrocytes, hippocampi of four WT pups were pooled and chopped. After the first centrifugation step, brain tissues were triturated in GGM (described above). Astrocytes were seeded in T75 flasks and maintained up to 8 weeks in culture. One day before dissection of neurons, 80,000 astrocytes were seeded per well on 12-well plates with NMM. The next day, 60,000 neurons were plated on 18-mm glass coverslips pre-coated with PLL. After 4 h, coverslips were inverted face down over the feeder layer with wax dots in between (as spacers between dish and coverslip to separate the two cell types). Every 3 days, half of the media was exchanged.

#### Aβ Preparation and Treatment of Primary Neurons

Treatment with synthetic Aβ_42_ peptides (GenicBio Synthetic Peptide) has been described earlier [79]. Briefly, Aβ_42_ was dissolved in DMSO to the final concentration of a 2 mM stock solution and aliquots were stored at − 80 °C and thawed immediately before use. On day 15 post-dissection, primary neurons on coverslips (see above) were separated from the astrocyte feeder culture and treated with a final concentration of 5 μM monomeric Aβ_42_ for 12 h at 37 °C in the cell culture incubator.

### Immunofluorescence Analysis of Hippocampal Primary Neurons and N2a Cells

Media supernatant was aspirated from neurons plated on 18-mm coverslips, and cells were washed with PBS, fixed with PFA solution (4% PFA, 4% sucrose in PBS), and then incubated for 10 min at room temperature (RT) on a shaker. After three washes with PBS, permeabilization with 0.25% Triton X-100 (in PBS) was performed for 10 min at RT. Following three additional washes with PBS, blocking (with 1% BSA + 0.25% Triton X100) was done for 1 h. Coverslips were incubated with primary antibodies against synaptophysin and MAP2 overnight at 4 °C while gently shaking. Next day, coverslips were washed three times with PBS and incubated with a fluorescent secondary antibody for 1 h at RT in the dark. All antibodies are listed in Suppl. Table 1A.

For quantification of dendritic spine density, primary neurons were obtained from two newborn mice per genotype. At least three images (i.e., three neurons) per mouse were taken by confocal laser scanning microscopy (TCS SP5, Leica). The gain settings were kept constant for all images acquired from the same experiment. Thereafter, TIFF images with merged channels were analyzed to measure synaptic punctae for selected regions of interest (3 to 5 dendritic segments per neuron) using SynPAnal software with a semi-automated puncta detection feature following a published protocol [80]. Puncta density values were used for the quantifications.

To study localization of transgenic N1, PrP-depleted (PrP-KO) N2a cells (see below) grown on coverslips were assessed 48 h after transfection (see below). Cells were washed with PBS, fixed for 15 min at RT with 4% PFA (in PBS), and again washed with PBS. Cells were blocked and permeabilized for 1 h in 10% FBS/0.1% glycine/0.1% saponin and incubated with primary antibodies in 1%FBS/0.1% glycine/0.1% saponin for 1 h at RT. Following three washes with PBS, incubation with secondary antibodies (in 1%FBS/0.1% glycine/0.1% saponin) was for 30 min. Additional washes with PBS were followed by mounting onto object slides using DAPI Fluoromount-G (Southern Biotech). Analysis was performed at a Leica TCS SP5 confocal microscope.

### N2a Cell Culture, CRISPR-Cas9 Genome Engineering, Transfections, and Treatments

To generate a PrP knockout in murine neuroblastoma cells (N2a; ATCC® CCL-131™), we used the CRISPR-Cas9 system described by Zhang et al. [81]. Targeting sequences were designed using the Web-based tool CRISPR Design (http://crispr.mit.edu/). The following target sequences directed to exon3 of the murine *Prnp* gene were used: sgRNA1_mPrP, ATTTTGCAGATCAGTCATCA; sgRNA3_mPrP, TCCTGATCGTGGGATGAGGG. The DNA sequences were synthesized (Sigma-Aldrich) and separately introduced into the plasmid vector pSpCas9(BB)-2A-Puro V2.0 (PX459; gift from Feng Zhang; Addgene plasmid # 62988). The N2a cell line was maintained at 37 °C under an atmosphere of 5% CO_2_ in Dulbecco’s modified Eagle’s medium (DMEM) + GlutaMAX™ (Life Technologies) with 10% (*v*/*v*) fetal calf serum (FCS; Sigma-Aldrich) and 100 U/mL penicillin, 100 μg/mL streptomycin. All cells were free of mycoplasma. Cells were transfected with the recombinant plasmids using Lipofectamine 2000 (Life Technologies). Twenty-four hours post-transfection, cells were selected with 3 μg/mL puromycin for 3 days. Single-cell clones were then cultured with normal culture medium, followed by screening for genetic modifications in *Prnp* by PCR amplification and direct sequencing (GATC-Biotech) with the following primers: mPrP_for, ACCTTCAGCCTAAATACTGG; mPrP_rev, AGCAACTGGTCTACTGTACAT. The absence of protein was confirmed by immunoblotting.

To express PrP, N1, or N1 fusion proteins, cells were transfected with the respective constructs using Lipofectamine 2000 following the manufacturer’s instructions. The antibody treatment of WT N2a cells was performed by adding 4 μg of either POM1 or 6D11 to 1 mL media supernatant (fresh OptiMEM) for 18 h.

### Biochemical Assessment of Cells, Conditioned Media, and Mouse Brains

N2a cells or primary neurons were washed with cold PBS and lysed using RIPA buffer (with Complete EDTA-free protease (PI) and phosphatase (PhosStop) inhibitor cocktails (Roche)), incubated on ice for 15 min prior to centrifugation at 12,000*g* for 10 min at 4 °C. For SDS-PAGE, cell lysates were mixed with Laemmli buffer plus 5% β-mercaptoethanol and denatured for 5 min at 95 °C.

For the analysis of the media supernatants (of N2a cells or primary neurons), experiments were carried out with freshly exchanged serum-free media (OptiMEM) incubated overnight. Supernatants were precipitated with trichloroacetic acid (TCA). For this, supernatants were collected and immediately incubated on ice with dissolved 10× concentrated protease inhibitor cocktail, cleared from dead cells and debris by consecutive mild centrifugations at 500*g* and 5000*g* for 5 min each. A total of 1/100 volume of 2% sodium deoxycholate (NaDOC) was then added to each sample. After 30-min incubation on ice, samples were mixed with 1/10 volume of 100% TCA and again incubated for 30 min on ice. After centrifugation at 15,000*g* for 15 min at 4 °C, the supernatant was aspirated and then air-dried for 5 min. The pellet was completely resuspended in 100 μL of 1× Laemmli buffer (incl. 5% β-ME) and boiled for 5 min at 95 °C.

Fresh or frozen brain tissue from TgN1 or WT littermates was used to prepare 10% (*w*/*v*) homogenates in RIPA buffer (50 mM Tris-HCl pH 8, 150 mM NaCl, 1% NP-40, 0.5% Na-Deoxycholate, 0.1% SDS) freshly supplemented with PI and PhosStop (except for the samples used for PK digestion) on ice. Samples were homogenized 30× using a Dounce homogenizer and incubated on ice for 15 min, shortly vortexed and incubated for another 15 min prior to centrifugation at 12,000*g* at 4 °C for 10 min. Total protein content was assessed by Bradford assay (BioRad). Supernatants were either further processed for SDS-PAGE or stored at − 80 °C.

For assessment of PrP^Sc^ levels in prion-infected mouse brains, 20% homogenates (*w*/*v*) of frontal brain were prepared in RIPA buffer without protease inhibitors. Again, samples were smashed 30× on ice using a Dounce homogenizer and subsequently spun down at 2000*g* for 2 min. The resulting supernatant was collected and 2 μL was digested with 20 μg/mL PK (Roche) in a total volume of 22-μL RIPA buffer for 1 h at 37 °C under mild agitation. Digestion was stopped by adding 6 μL of 4× Laemmli buffer (incl. 5% β-ME) and boiled for 5 min at 95 °C. Subsequent SDS-PAGE and Western blot analysis was performed as described above.

For SDS-PAGE, denatured samples were loaded on either precast Nu-PAGE 4–12% Bis-Tris protein gels (Thermo Fisher Scientific) or Any kD™ Mini-PROTEAN® TGX™ Precast Protein Gels (BioRad). After electrophoretic separation, proteins were transferred to nitrocellulose membranes (BioRad) by wet-blotting and membranes were subsequently blocked for 30 min with 1× RotiBlock (Carl Roth) in TBS-T and incubated overnight with the respective primary antibody (Suppl. Table 1) diluted in either 5% BSA or 1× RotiBlock in TBS-T at 4 °C on a shaking platform.

Nitrocellulose membranes were subsequently washed with TBS-T and incubated for 1 h at RT with either HRP-conjugated secondary antibodies or in the dark with secondary antibody conjugates IRDye® 680RD and 800CW (Li-Cor) and subsequently washed 5× with TBS-T. For classical chemilumiscence detection, after incubation with Pierce ECL Pico or Super Signal West Femto substrate (Thermo Fisher Scientific), signal was detected with a ChemiDoc imaging station (BioRad). The fluorescence signals were detected using an Odyssey CLX system (Li-Cor). Densitometric quantification was done using the Image studio lite version 5.2.

### Real-Time Quantitative Polymerase Chain Reaction

Mouse brain tissues from forebrain or cerebellum (about 100 mg) were collected and homogenized with 1 mL TRIzol and incubated for 5 min at RT to permit the complete dissociation, and then centrifuged at 12,000*g* for 10 min at 4 °C. The upper phase was collected after centrifugation and 200 μL chloroform were added to each sample and vigorously shaken by hand for 15 s and incubated at RT for 2–3 min. The upper aqueous phase was collected after centrifugation of the mixture at 12,000*g* for 15 min at 4 °C and, subsequently, 500 μL of 100% isopropanol were added to the collected aqueous phase and incubated for 10 min at RT. After another centrifugation step, the RNA pellet was washed twice with 1 mL of 75% ethanol, vortexed and centrifuged at 7500*g* for 5 min at 4 °C. After air-drying for 5–10 min, the pellet was dissolved in DEPC water and heated at 55 °C for 10 min. Purity and concentration of RNA were assessed by NanoDrop™ measurement (Thermo Scientific). The cDNA was synthesized using reverse transcriptase with a two-step method using RevertAid H Minus First Strand cDNA Synthesis Kit (Thermo Scientific). Every reagent was added according to the manufacturer’s instructions. Three replicates were set for each group. The ribosomal protein L13 (RPL13) was used as the reference gene, and the relative expression level of PrP was calculated by the 2^−ΔΔCt^ method [82]. Primers are listed in Suppl. Table 1B.

### Histological Assessment

Morphological analysis was performed as described previously [83]. After collecting the brains, they were fixed in 4% paraformaldehyde (PFA) overnight. In the case of prion- or mock-inoculated animals, samples were initially inactivated for 1 h in 98–100% formic acid before being exported from the respective biosafety facility. After several washes with water, samples were again incubated overnight with 4% PFA at 4 °C. Afterwards, samples were embedded in low melting point paraffin according to the standard laboratory procedures. Sections with 4 μm thickness were prepared and stained either with hematoxylin and eosin (HE) or following standard immunohistochemistry procedures using the Ventana Benchmark XT machine (Ventana, Tucson, AZ). Briefly, for antigen retrieval, deparaffinated sections were boiled for 30–60 min in 10 mM citrate buffer (pH 6.0). All primary antibodies (see Suppl. Table 1A) were prepared in 5% goat serum (Dianova, Hamburg, Germany), 45% Tris buffered saline with 0.1% Triton X-100 (TBST) pH 7.6, in antibody diluent solution (Zytomed, Berlin, Germany). Detection was performed with anti-rabbit or anti-goat histofine Simple Stain MAX PO Universal immunoperoxidase polymer or Mouse Stain Kit (for detection of mouse antibodies on mouse sections). All secondary antibody polymers were purchased from Nichirei Biosciences (Tokyo, Japan). For detection of antibodies, Ultra View Universal DAB Detection Kit or Ultra View Universal Alkaline Phosphatase Red Detection Kit from Ventana were used according to standard settings of the machine. Experimental groups were stained all at the same time to provide identical conditions. For PrP^Sc^ staining, pre-mounted tissue sections with 4-μm thickness were first treated with 98–100% formic acid for 5 min and further processing was performed with the above-mentioned automated staining machine. Briefly, sections were pretreated with 1.1 mM sodium citrate buffer (2.1 mM Tris-HCl, 1.3 mM EDTA, pH 7.8) at 95 °C for 30 min, digested with low concentration of PK for 16 min, incubated first in Superblock for 10 min and then with the PrP-specific antibody SAF84, followed by secondary antibody treatment and detection.

### Statistical Analysis

Statistical analysis of Western blot results, morphological quantifications of dendritic spines, and qRT-PCR results between experimental groups was performed using Student’s *t* test and assessment of significance for incubation times after prion inoculation was performed using log-rank (Mantel-Cox) test for two-group comparisons with consideration of statistical significance at *p* values < 0.05 (*), < 0.01 (**), and < 0.001 (***).

## Results

### Generation and Characterization of TgN1 Mice: Transgenic Overexpression of N1 Does Not Result in Obvious Phenotypic Alterations

**Fig. 2:**
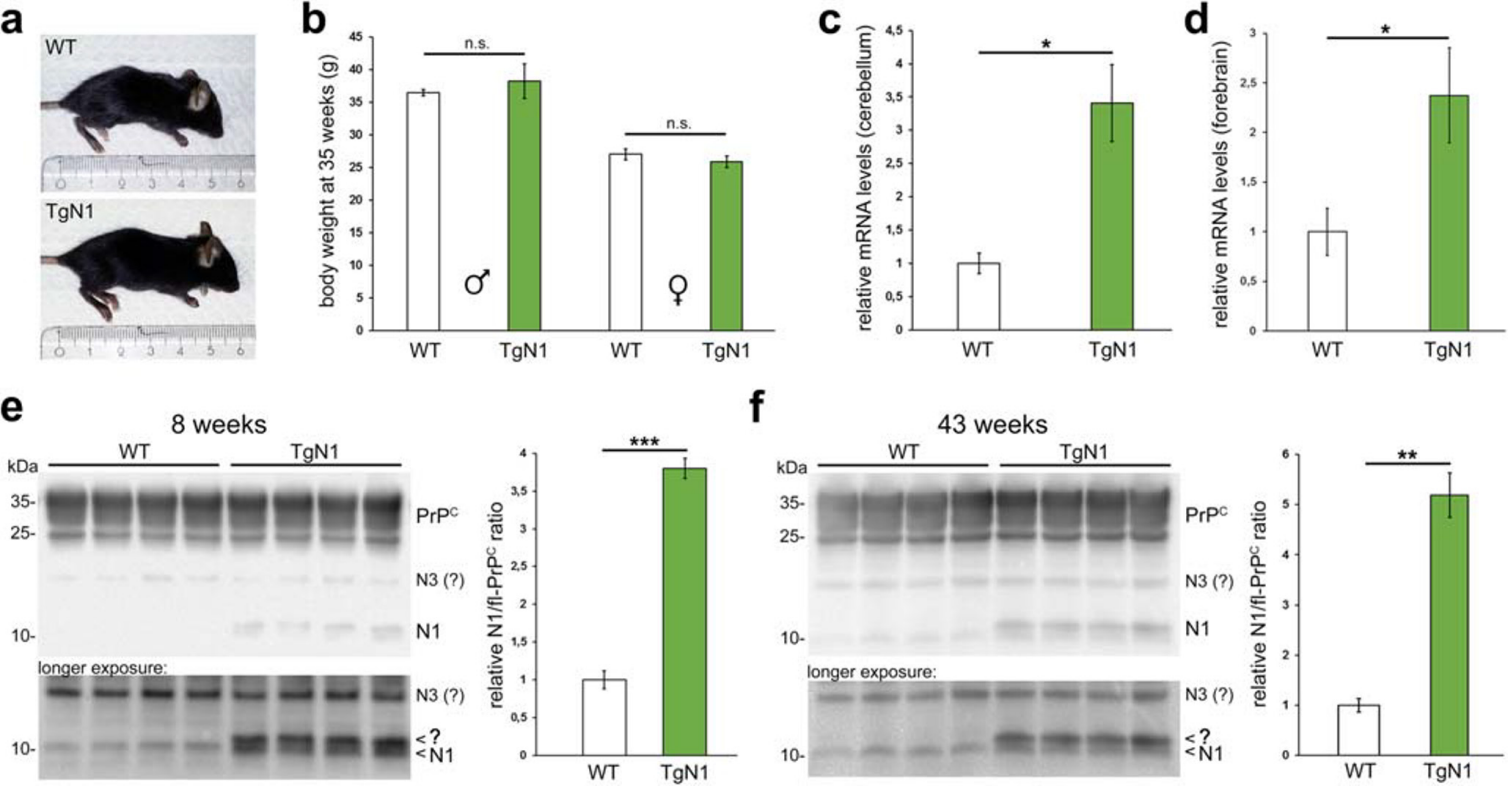
Characterization of TgN1 mice. Body size (**a**) and body weight measurements (**b**; *n* = 3) reveal no significant differences between TgN1 and the age-matched WT littermates. **c**, **d** RT-qPCR analysis in mouse brain homogenates showing higher mRNA expression levels in TgN1 compared to WT controls in cerebellum (**c**; *n* = 3; *p* = 0.031) and forebrain (**d**; *n* = 3; *p* = 0.028). (**e**, **f**) Western blot analyses of forebrain samples of **e** 8-week-old (*n* = 4; *p* = 0.00001) and **f** 43-week-old mice (*n* = 4; *p* = 0.0047) (measured as ratio of N1 fragment to corresponding fl-PrP; both detected by POM2 antibody) reveal a double band (arrowheads; upper band indicated by a “?”) and a strong increase in the levels of N1 fragments in TgN1 mice (note that in the case of TgN1 the double band was measured as “N1”). Taken together, these data demonstrate successful generation of transgenic mice overexpressing N1. In addition, a fragment of ~ 20 kDa, possibly representing the endogenous N3 fragment resulting from the γ-cleavage of PrP^C^, is readily detected in brain homogenates

The released, soluble N1 fragment of PrP^C^ has been linked to neuroprotective functions in neurodegenerative conditions; yet, the protease responsible for its endogenous production remains obscure. Therefore, to directly assess the role of N1 in prion diseases, we generated transgenic mice (TgN1) stably overexpressing N1 under the control of the prion protein promotor (using the half genomic construct [77]) on a wild-type (C57Bl/6) background (as expression of PrP^C^ is a prerequisite for prion disease [84]). These mice neither presented any obvious behavioral alterations nor differences in size (Fig. 2a) or body weight (*n* = 3; SEM; Fig. 2b) when compared to wild-type (WT) littermate controls. Overexpression of the transgene was confirmed on the genetic level by copy number analysis (ΔΔCt = − 3.913, corresponding to a fold change of 15; data not shown) and on the mRNA level for cerebellum (TgN1: 3.41 ± 0.58; WT set to 1.00 ± 0.15; *n* = 3; SEM; Fig. 2c) and forebrain (TgN1: 2.37 ± 0.48; WT set to 1.00 ± 0.24; *n* = 3; SEM; Fig. 2d). Note that primers used for RT-qPCR bind to regions coding for the N-terminal part of PrP^C^ and therefore do not per se discriminate the N1 transgene from endogenous *Prnp*. Most importantly, western blot (WB) analysis of freshly prepared forebrain homogenates clearly proved overexpression of N1 (in 8-week-old mice: 3.8 ± 0.14 for TgN1; WT set to 1.00 ± 0.12; *n* = 4; SEM; Fig. 2e) that was even more pronounced in aged mice (at 43 weeks: 5.2 ± 0.44 for TgN1; WT set to 1.00 ± 0.14; *n* = 4; SEM; Fig. 2f), whereas levels of full-length PrP^C^ and an N-terminal PrP fragment of ~ 20 kDa (possibly corresponding to N3 resulting from the recently described γ-cleavage [85]) were not altered between genotypes. However, in TgN1 mice, our biochemical analysis also revealed the presence of a conspicuous double band at ~ 11 kDa, i.e., the expected molecular weight of N1 (indicated by a question mark in Fig. 2e, f). To better clarify the nature of those N-terminal fragments, we performed a differential analysis of mouse brain homogenates by comparing bands detected with two N-terminally binding antibodies with the ones obtained with two C-terminally directed antibodies (Fig. 1; Suppl. Fig. 1a) and size comparison with recombinant N1 (Suppl. Fig. 1b). These analyses provided further support that the ~ 20 kDa band is obviously N3. Importantly, they let us to conclude that the band running slightly higher than bona fide N1 most likely represents N1 with an uncleaved N-terminal signal peptide (N1-SP), which will be further discussed below.

Since it is conceivable that transgenic N1 expression could influence the endogenous α-cleavage of PrP^C^, we compared levels of the C1 fragment in brain homogenates of TgN1 and WT mice (Suppl. Fig. 1c). As no significant differences were found, no feedback effect of transgenic N1 on the α-cleavage rate seems to be present.

Next, we investigated whether key candidate signaling pathways associated with PrP^C^ or N1 were influenced by transgenic overexpression of N1 in 43-week-old mice. Upon biochemical assessment, no alterations in the ratio of phosphorylated and total levels of protein kinase B (Akt), eukaryotic initiation factor 2 (eIF2α), or MAP kinases Erk1/2 and p38 could be detected in forebrain homogenates (Suppl. Fig. 2).

**Fig. 3:**
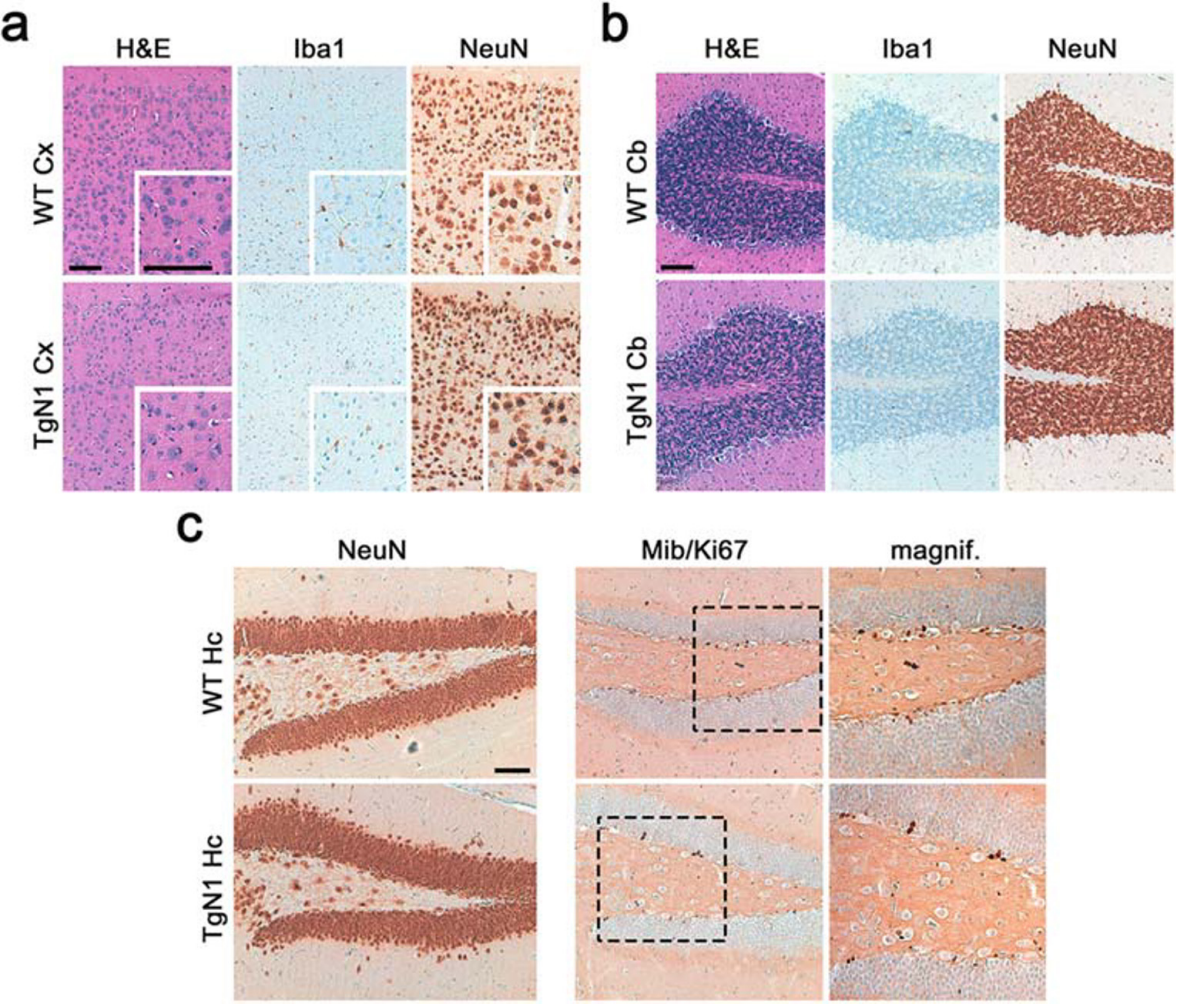
Lack of overt morphological alterations in young TgN1 mice. **a**, **b** Neither H&E staining nor immunohistochemical detection of microglia (Iba1) and neurons (NeuN) revealed any alterations between eight-week-old WT and TgN1 mice in cortical (Cx; **a**) or cerebellar brain regions (Cb; **b**). **c** Likewise, neuronal density (assessed by neuronal marker NeuN) and amounts of proliferating cells (assessed by the marker Mib/Ki67) were similar between both genotypes in the hippocampus (Hc). Scale bars represent 100 μm

Lastly, (immuno)histochemical analyses did not reveal any neuropathological alterations in 8-week-old TgN1 mice when compared to WT littermates with regard to overall brain morphology, content and distribution of mature neurons, cellular proliferation and microglial activation in cortical (Fig. 3a), and cerebellar areas (Fig. 3b) as well as in hippocampus (Fig. 3c). For older mice, refer to Suppl. Fig. 3.

#### Increased PrP^Sc^ Levels and p38 MAP Kinase Activation but Unchanged Disease Course in Prion-Infected TgN1 Mice

**Fig. 4:**
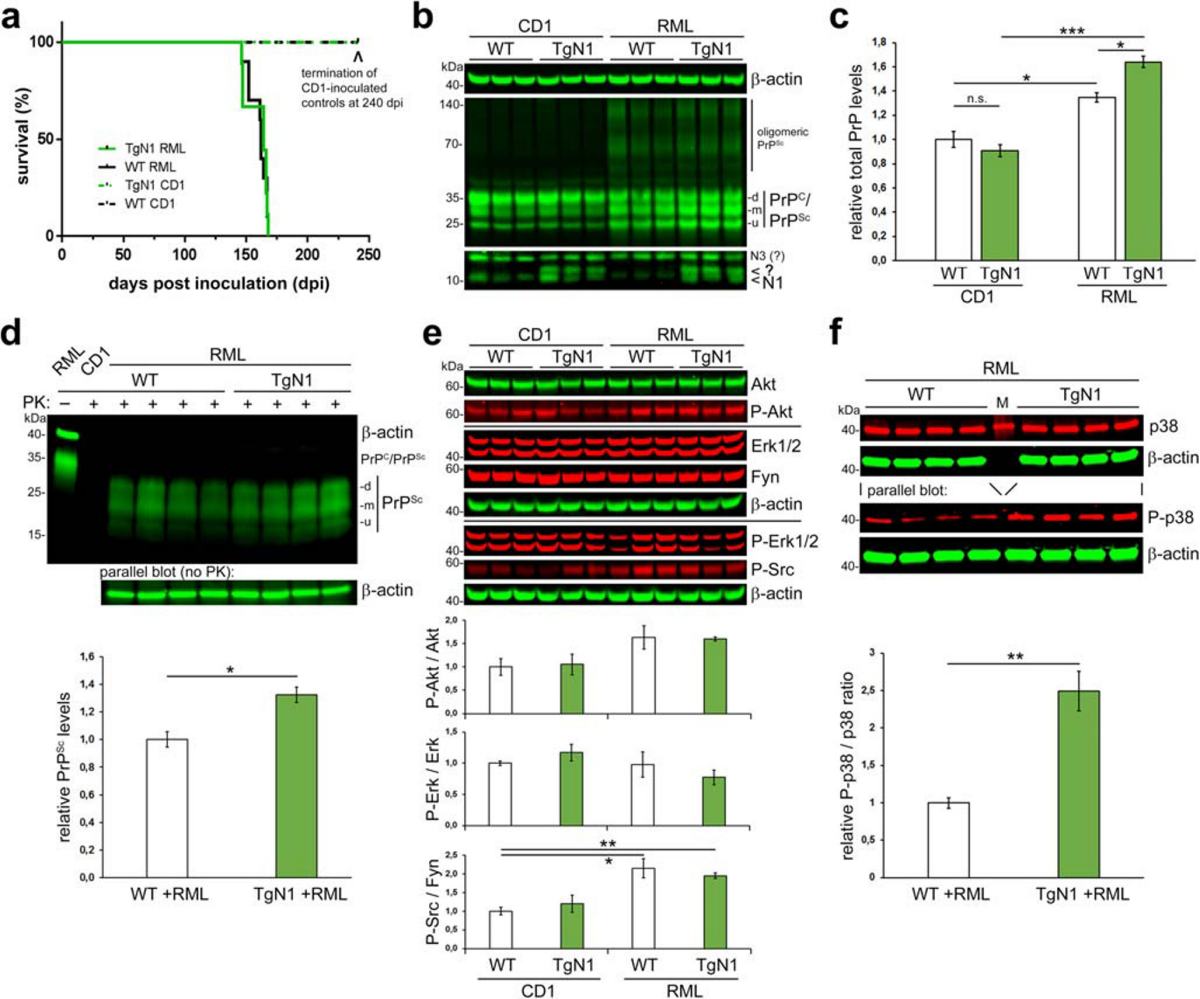
Intracerebral inoculation of TgN1 and control mice with prions. **a** Kaplan-Meier survival curve of mice upon intracerebral inoculation with mouse-adapted RML prions showing similar incubation times to terminal prion disease for TgN1 (*n* = 10) and WT littermates (*n* = 9), whereas the mock-inoculated control groups of each genotype (inoculated with CD1 brain homogenate) did not show any clinical signs until sacrification at 240 days post-inoculation (*n* = 4). **b** Western blot analysis of infected and non-infected mouse brain homogenates showing an altered glycopattern and presence of oligomeric PrP^Sc^ forms in prion-infected samples and increased total PrP levels (i.e., PrP^C^ and PrP^Sc^) in terminally diseased TgN1 mice (quantification in **c**; *p* = 0.0167; *n* = 3). **d** Western blot of PK-digested brain samples of terminally diseased mice of both genotypes (quantification was done by normalizing the PrP^Sc^ signals (POM1 antibody) against actin on the parallel blot with non-digested samples) (*n* = 4; *p* = 0.0167). Controls (on the left) include a non-digested RML-infected brain homogenate and a CD1-inoculated PK-digested brain sample. The shift in molecular weight and disappearance of the actin signal confirm successful enzymatic digestion. **e**, **f** Western blot analyses of candidate signaling pathways associated with prion disease showing **e** no differences in the phosphorylation state of Erk1/2, slightly elevated P-Akt and significantly increased activation of the P-Src in prion-infected mice (WT + CD1 vs. WT + RML: *p* = 0.027; WT + CD1 vs. TgN1 + RML: *p* = 0.003; *n* = 3) and **f** a significant increase in the activating phosphorylation of p38 in infected TgN1 samples compared to infected controls (*p* = 0.0032; *n* = 4)

After confirming expression of the transgene, we intracerebrally inoculated TgN1 mice and WT littermates with mouse-adapted RML. Unexpectedly, mice of both genotypes presented with an equal disease course reaching terminal disease at similar time-points (mean: TgN1 + RML: 160 ± 7 days; *n* = 10; WT + RML: 159 ± 9 days; *n* = 9; SD), whereas mice (of both genotypes) inoculated with a non-pathogenic control homogenate (CD1) were sacrificed without any clinical signs at day 240 (Fig. 4a). We next performed WB analyses of brain homogenates obtained from terminally prion-diseased and CD1-inoculated control mice of both genotypes (Fig. 4b). Again, a double band for N1 is apparent in TgN1 samples with the upper one (likely representing N1-SP) running slightly higher than endogenous N1 (in WT). Prion conversion in RML-infected mice of both genotypes was confirmed by (i) increased levels of total PrP species (i.e., PrP^C^ plus PrP^Sc^), (ii) a shifted glycopattern, and (iii) appearance of SDS-stable oligomeric PrP conformers when compared to CD1-inoculated samples (Fig. 4b, c). A surprising reduction in levels of endogenous N1 was detected in prion-diseased WT mice. Although this would require further analyses, it may indicate recruitment of N1 into insoluble PrP^Sc^ aggregates or reduced α-cleavage efficiency in prion disease. Of note, we found slightly but significantly higher levels of total PrP in infected TgN1 versus WT mice (TgN1 + RML: 1.63 ± 0.05; compared to WT + RML: 1.34 ± 0.04; with non-infected WT(+CD1) set to 1.00 ± 0.07; *n* = 3; SEM; Fig. 4c). This increase was likely due to higher PrP^Sc^ levels (TgN1 + RML: 1.32 ± 0.05; WT + RML set to 1.00 ± 0.06; *n* = 4; SEM) as assessed upon digestion of brain homogenates with proteinase K (PK) (Fig. 4d). We next investigated signaling pathways associated with PrP^C^ and prion diseases. While no relevant differences were found in the phosphorylation state of the MAP kinase Erk1/2, prion infection resulted in slightly elevated phosphorylation of Akt and significantly increased activation of the Src kinase Fyn (Fig. 4e). However, no differences were observed between genotypes. In contrast, we found a significant increase in the activating phosphorylation of the MAP kinase p38 in infected TgN1 mice compared to infected controls (TgN1 + RML: 2.49 ± 0.26; WT + RML set to 1.00 ± 0.07; *n* = 4; SEM; Fig. 4f). This difference between genotypes was not observed in non-infected mice (Suppl. Fig. 2).

Histological analysis of neuropathological hallmarks, such as astrogliosis, microglia activation, and spongiosis, confirmed fully established prion disease in RML-infected compared to CD1-inoculated mice, yet did not reveal any overt differences between genotypes (Suppl. Fig. 3).

In summary, transgenic N1 overexpression did not result in protection against prion disease. By contrast, PrP^Sc^ levels and p38 MAPK signaling were even increased in terminal TgN1 mice yet did not alter the clinical course and incubation time.

#### Transgenically Expressed N1 Does Not Protect Against Aβ-Mediated Synaptotoxicity

**Fig. 5:**
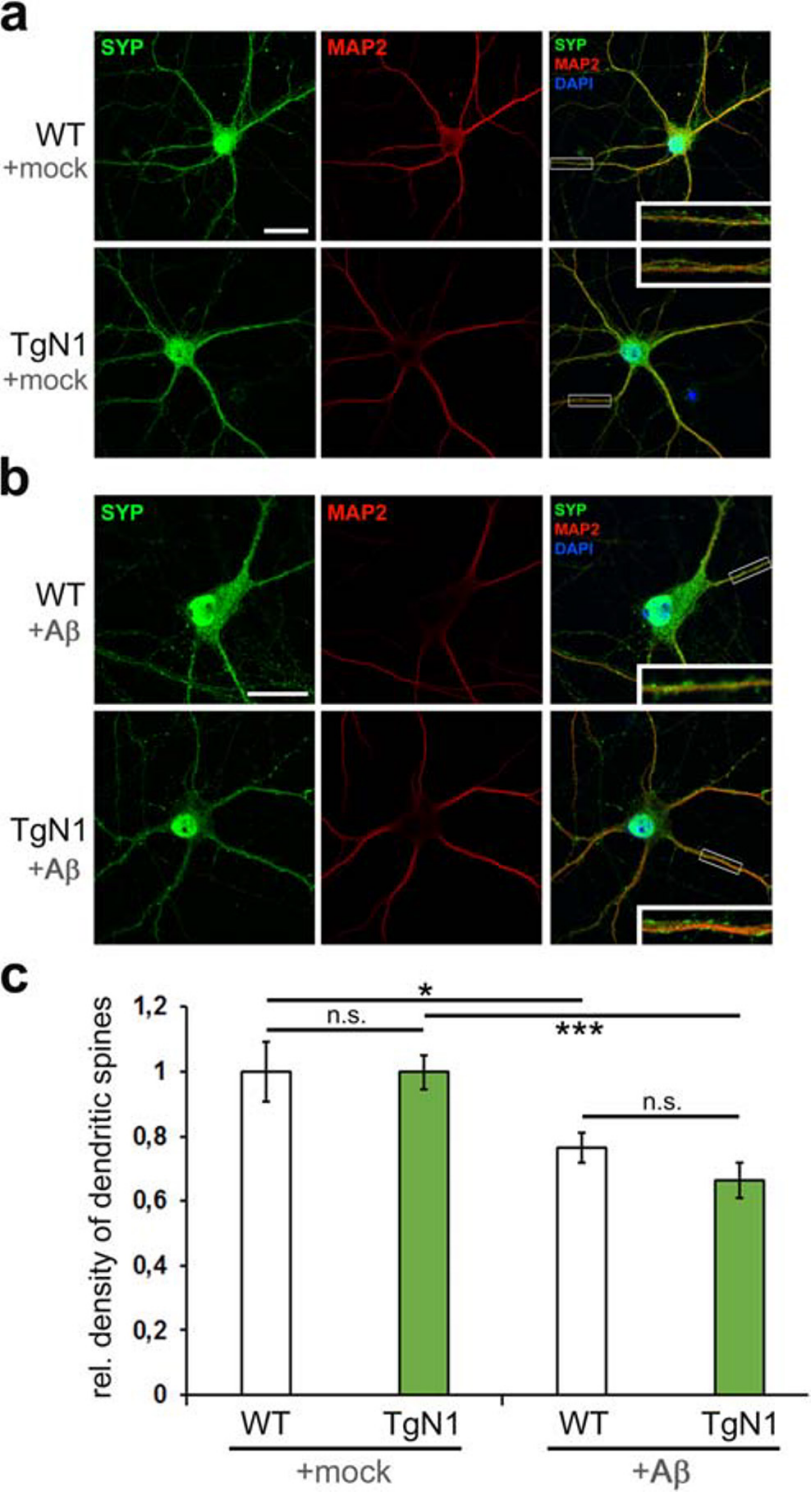
Morphological assessment and Aβ_42_ treatment of primary neurons derived from TgN1 and control mice. **a** Representative images from primary neurons grown in a co-culture system conditioned with an astrocyte feeder layer for 2 weeks, indicating no difference in overall morphology and dendritic spines density (green dots) between TgN1 and WT neurons (quantification in **c**). SYP = synaptophysin (green); MAP2 = microtubule-associated protein 2 (red). **b**, **c** Treatment of neurons with synthetic Aβ_42_ resulted in a decrease in dendritic spine density compared to mock-treated controls (**a**, **c**); yet, no differences were found between treated neurons of both genotypes (**b**, **c**). Scale bar is 25 μm

N1 was shown to bind to Aβ in the extracellular space and to protect neurons from Aβ-associated toxicity [38]. Synaptotoxicity, which precedes neuronal loss in neurodegenerative conditions, can be assessed by quantification of dendritic spines [30]. To study the effects of transgenic N1 overexpression, primary neurons were obtained from TgN1 mice and WT littermates and maintained in co-culture with an astrocyte feeder layer. Microscopic analysis (Fig. 5) revealed no differences in overall morphology and relative density of dendritic spines for TgN1 (TgN1: 0.999 ± 0.052; WT set to 1.00 ± 0.092; SEM) between neurons of both genotypes when treated with solvent only (+mock; Fig. 5a, c). Thus, transgenic overexpression of N1 does not cause alterations in neuronal morphology or dendritic spine density. Upon 12 h of treatment with synthetic Aβ_42_ (Fig. 5b, c), dendritic spine density was significantly reduced compared to mock-treated neurons (with “WT + Aβ”: 0.764 ± 0.047 and “TgN1 + Aβ”: 0.665 ± 0.055; SEM). However, there were no significant differences between Aβ-treated neurons from both genotypes. This indicates that, as observed above for prion diseases (Fig. 4), transgenic overexpression of N1 does not confer protection against exogenously administered proteopathic entities.

#### Impaired ER Translocation Results in Retention of Transgenic N1 Fragments (with Likely Uncleaved ER-Targeting Signal Peptide) in the Cytosol

To explain this lack of protection in spite of confirmed overexpression of the assumedly beneficial N1 fragment in the brains of TgN1 mice, we performed further analyses at the cellular level.

**Fig. 6:**
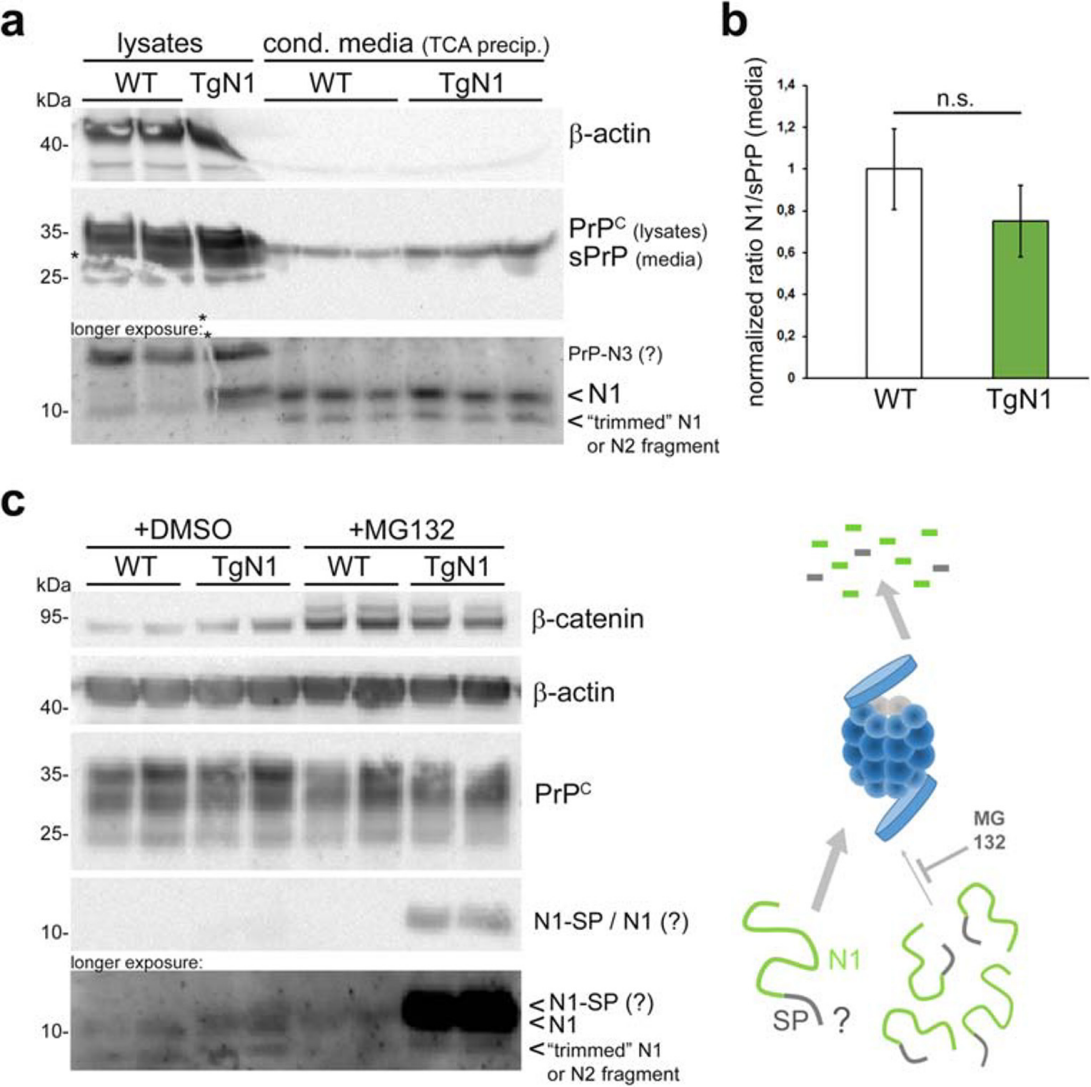
Transgenically expressed N1 is not or only poorly secreted and rather retained with uncleaved SP in the cytosol. **a** Lysates and conditioned media of primary neurons (in mono-culture) from WT and TgN1 mice were biochemically analyzed for N1 and PrP^C^. While overexpression of N1 is confirmed in lysates of TgN1 neurons (note the double band), no increase (instead rather a tendency towards decrease (**b**)) in N1 is found in the respective media supernatants. Note that a band lower than N1 is detected in media which may represent “trimmed” N1 (see Suppl. Fig. 4a) or endogenous N2 resulting from the β-cleavage of PrP^C^. **b** Densitometric quantification of **a**; *n* = 3; levels of N1 signals were referred to levels of shed PrP found in media (a band possibly corresponding to N3 was detected in lysates but not in supernatants). **c** Primary neurons treated with an inhibitor of the proteasome (+MG132) or diluent only (+DMSO; as control) followed by Western blot analysis of lysates for levels of N1 fragments, PrP^C^, β-catenin, and β-actin (note that the low biostability of N1 may lead to fast degradation explaining the occasional need for longer exposure. As mentioned above, a weak band running lower than 10 kDa might reflect N2 or “trimmed” N1). Block of the proteasome results in a strong increase of an N1 fragment—most likely—N1-SP (here appearing as just one strong band) in the cytosol (as indicated in the scheme on the right)

We first studied the secretion of N1 by primary neurons into the media supernatant by WB analysis. While overexpression of the transgene was confirmed in corresponding neuronal lysates (Fig. 6a), no such increase of N1 was observed in the conditioned media of TgN1 neurons (TgN1: 0.75 ± 0.17; WT set to 1.00 ± 0.19; *n* = 3; SEM; Fig. 6a, b), indicating that transgenic N1 was not (or only poorly) secreted but rather retained in the cells. This is in strong agreement with cell culture studies revealing impaired translocation of IDDs into the ER [6, 86, 87]. Accordingly, transgenic N1 (with unprocessed N-terminal signal peptide (SP)) would be retained in the cytosol. To study this, we inhibited proteasomal degradation and performed WB analysis (Fig. 6c). As expected, this proteasomal inhibition (confirmed by elevated levels of β-catenin) increased N1 fragments in the cytosol of TgN1 neurons, supporting the concept that transgenic N1 is not (efficiently) imported into the ER but rather stays in the cytosol. This probably also explains the presence of the double band (lower band: N1 (~ 10 kDa); upper band: N1 with uncleaved signal peptide (N1-SP ~ 12 kDa)) observed in brain samples of TgN1 mice throughout this study (Figs. 2e, f and 4b) and reveals for the first time that ER translocation and secretion of IDDs is also impaired in vivo.

**Fig. 7:**
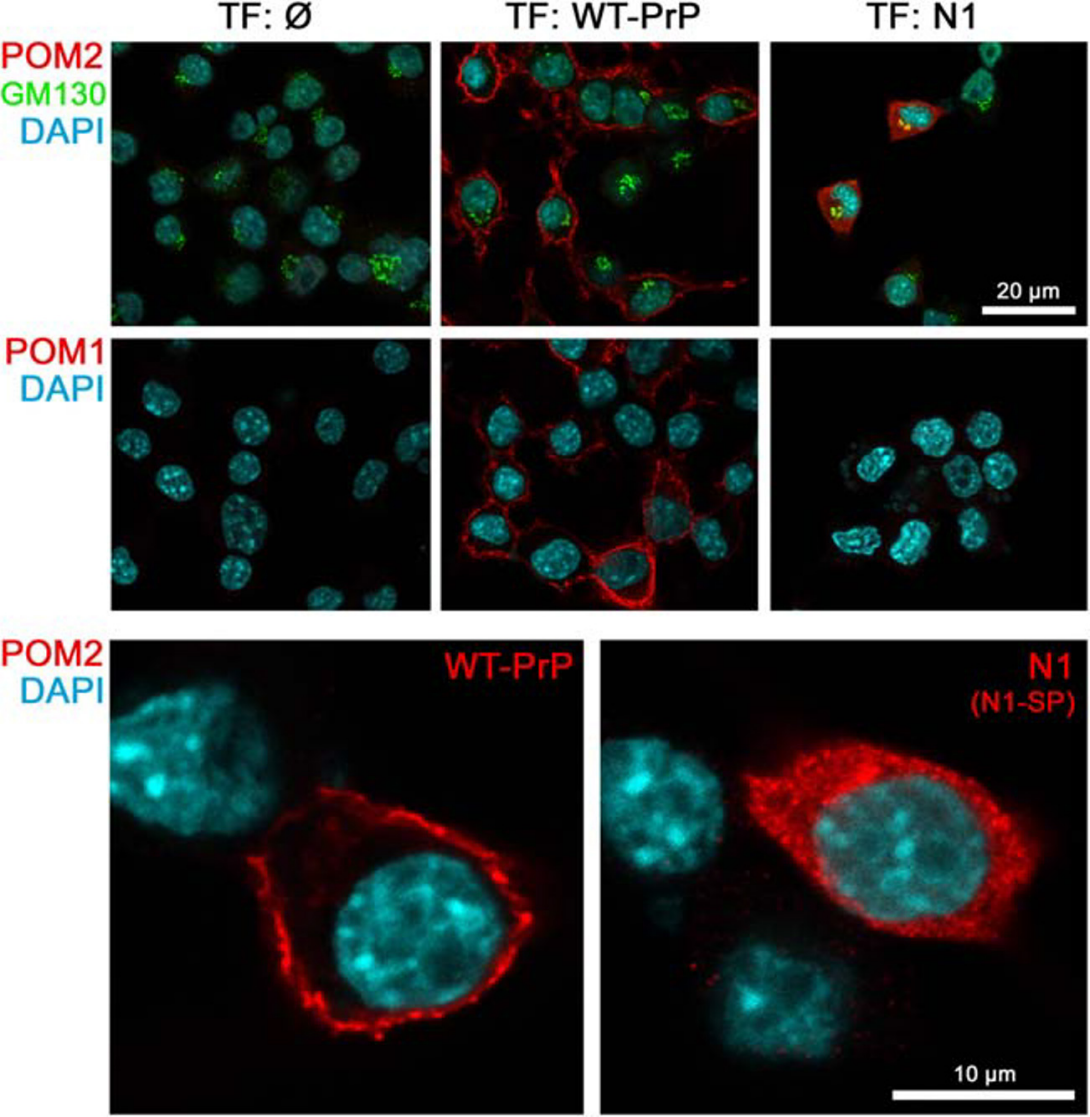
Cytosolic retention of ectopically expressed N1. PrP-KO N2a cells were transfected with plasmids coding for either WT-PrP or N1 alone. Non-transfected cells served as negative controls. Immunfluorescent stainings on fixed and permeabilized cells revealed a predominant membrane-staining for WT-PrP with both PrP-directed antibodies (POM2 and POM1, red), whereas cells transfected with N1 showed a strong cytosolic staining pattern (note that POM1 does not detect N1; specificity control). GM130 (green) = Golgi marker. Scale bars represent 20 μm (upper panel) or 10 μm (lower two pictures)

To further study the cellular localization of ectopically expressed N1, we transiently transfected N2a cells devoid of endogenous PrP (PrP-KO N2a cells). In stark contrast to WT-PrP, which was mainly found at the plasma membrane, cells expressing N1 alone revealed a strong cytosolic staining (Fig. 7). In conclusion, biochemical data showing (i) a TgN1-restricted double band with the upper band running higher than bona fide N1, (ii) accumulation of that fragment upon proteasomal inhibition, and (iii) impaired secretion of transgenic N1, together with (iv) a morphological analysis revealing cytosolic localization strongly suggest that transgenic N1 is retained inside cells with an uncleaved SP.

#### Aspects to Be Considered When Devising Improved Transgenic Mouse Models for N1-Based Therapeutic Approaches

To better understand the intricate nature of different N1 forms found in biochemical analyses [48], we finally performed additional experiments in murine neuroblastoma (N2a) cells. We and others have repeatedly experienced difficulties in the reliable detection of endogenous, proteolytically generated N1, which may result from its low biostability, fast degradation upon release [54], and general “stickiness” (e.g., towards plastic surfaces; recent own observation) among others. In WB analyses of conditioned media as well as in cell lysates and brain homogenates that had undergone freezing and thawing (e.g., Fig. 6a, c), but not in freshly prepared brain homogenates or cell lysates (e.g., Figs. 2e, f or 4b), “N1” presented with several bands of slightly lower molecular weight than bona fide N1 [59, 88], indicative of a proteolytic trimming event. To further assess this, we analyzed endogenously produced N1 in conditioned media of N2a wild-type cells. While fragmentation of N1 was again observed in control-treated overnight cultures, treatment of cells with an antibody against the epitope ranging from amino acid 93 to 109 (corresponding to the C-terminal end of N1) completely blocked this trimming (Suppl. Fig. 4a). This finding demonstrates that N1, once released (either by physiological α-cleavage or by disruption of cells and tissue), is stepwise truncated from its C-terminus assumingly through unspecific cleavage by extracellular proteases. This may explain N1’s low biostability and, given that the trimming affects one of N1’s described Aβ-binding sites (aa 95–110 [36]), indicates the need of a stabilizing C-terminal modification when considering treatment options based on exogenous administration of N1 derivates. Moreover, this trimming impairs binding of certain antibodies with epitopes in this region, such as the commercially available 6D11 and 3F4 antibodies. Lastly, it may be considered to generate fragments mimicking bona fide N2 resulting from the PrP^C^ β-cleavage.

It has been described that, for ER translocation of a given peptide sequence to be successful, structural elements, such as α-helical domains, have to be present in the nascent chain [87, 89]. In the case of PrP^C^, these criteria are fulfilled by its structured C-terminal half (Fig. 1) [86]; yet, transgenically expressed N1 is lacking these elements and is therefore retained in the cytosol (as shown here in mice and earlier in vitro [6]). To overcome this problem and to allow for efficient N1 secretion and increased biostability (by protection from the C-terminal trimming mentioned above; Suppl. Fig. 4a), we expressed N1-fusion proteins harboring either the Fc region of an IgG (N1Fc) or a nanobody (N1Nb) C-terminal of N1 in PrP-depleted N2a cells. As expected [38], in contrast to N1 alone (of which only low amounts were detected in media thus reflecting its impaired translocation and secretion), both N1Fc and N1Nb were efficiently secreted and could be readily detected in conditioned media (Suppl. Fig. 4b). Of note, in addition to the respective full-length fusion proteins, we also detected a lower band likely corresponding to bona fide N1. This indicates that α-cleavage (or an α-cleavage-like proteolytic event) still occurs, even if (not necessarily membrane-anchored) structure-lending protein tags replace the C-terminal half of PrP^C^. This further highlights the extreme tolerance of this cleavage event described earlier [72].

## Discussion

An increasing body of evidence indicating protective effects of PrP-N1 in neurodegenerative conditions let us to generate transgenic mice overexpressing this fragment and enabling detailed studies in animals. However, likely due to the lack of α-helical elements in the nascent chain, transgenically expressed N1 was not or only inefficiently translocated into the ER and, hence, not or poorly secreted into the extracellular space but rather retained (seemingly with the uncleaved N-terminal signal peptide (N1-SP)) in the cytosol. As such, our study is the first mouse model to illustrate the requirements of structured domains in secretory proteins for efficient ER translocation, as shown previously in cultured cells [6, 86, 87, 89]. Accordingly, though the N-terminal ER-targeting SP is necessary [90, 91], it is not sufficient to bestow efficient translocation for all proteins determined to be secreted. In the case of largely unstructured proteins, additional structural elements (i.e., α-helical domains) are required at one point in the growing peptide chain in order to be translocated.

Consequently, C-terminal addition of an IgG Fc part (as already shown earlier [38]) or a nanobody rescued N1 secretion in our experiment. Although transgenic N1 was not or only poorly secreted but aberrantly located in the cytosol, our study provides relevant insight into different aspects of prion pathophysiology and reveals aspects to be considered for future studies on N1-based treatment approaches. Along our analyses, we also found indications that the N3 fragment resulting from the γ-cleavage of PrP^C^ [85] can be readily detected in normal mouse brain and may thus bear physiological relevance.

### No Protection of Cytosolic N1 Against Proteopathic Seeds

Presence of aberrant subcellular forms, including cytosolic PrP (cytPrP), has been described decades ago [92–96]. CytPrP may result from pathogenic mutations, retrotranslocation from the ER in various conditions of cellular stress, or from inefficient ER translocation [97–99] and is found under physiological conditions in neurons of different brain regions [100]. Aggregation-prone cytPrP is constitutively cleared by the proteasome; yet, impairment of this degradation results in cytoplasmic accumulation [97, 98, 101]. Fittingly, upon proteasomal inhibition, we also observed drastic accumulation of N1 with uncleaved SP in TgN1 primary neurons.

To date, there is still some controversy regarding putative pathophysiological roles played by cytPrP. While some studies found cytPrP accumulation to cause neurotoxicity and cell death per se [102–104], others found no adverse effects and rather indicated cell-protective activities [105–107]. Likewise, the contribution of cytPrP to the misfolding and neurotoxicity in prion diseases still remains enigmatic. Though cytPrP expressed in *Drosophila* was not toxic per se, it contributed to the detrimental effects when exposed to prions [108]. Several other studies have also pointed towards a harmful role of cytPrP in prion diseases [102, 109, 110]. At least two—not mutually exclusive—paradigms of prion-associated toxicity are currently being put forward by supportive experimental data: a model of “external toxicity” emphasizes binding of extracellular prions (and other proteopathic seeds, such as Aβ oligomers) to surface PrP^C^ inducing neurotoxic signaling cascades and/or membrane perturbations [3, 31, 36, 38, 111–113], whereas another concept describes detrimental effects of intracellular (including cytoplasmic) prions abrogating essential homeostatic and degradative processes of the cell including the ubiquitin-proteasome system (UPS) [102, 114–119]. While our study did not directly address the importance of cytosolic PrP species in prion diseases, lack of effects in our transgenic mice with high N1 (or better: N1-SP) levels in the cytoplasm (as demonstrated in N1-transfected cells) at least indicates that N1 does not confer protection against cytPrP-mediated toxicity or cytosolic prions.

In contrast to any protective effects, the only difference found between our transgenic and control mice upon prion infection was a moderate increase in forebrain PrP^Sc^ levels in TgN1. Given that the proteasome is involved in PrP^Sc^ degradation [102, 114, 120–122], it is conceivable that the massive overproduction of cytosolic N1 to some extent reduced the efficiency of the proteasome to degrade PrP^Sc^. And since activation of the MAP kinase p38 has been specifically linked to the toxic signaling underlying prion diseases [113, 123, 124], it seems likely that the increase in p38 phosphorylation in our TgN1 mice at terminal prion disease is a consequence of the elevated PrP^Sc^ levels.

In AD, cellular uptake of Aβ occurs and intraneuronal accumulation of Aβ has been linked to Aβ-induced neurotoxicity [125]. In our experiments, cytoplasmic N1 did neither confer protection against prions nor against Aβ-induced neurotoxicity. Since exogenously administered N1 reliably blocks Aβ-induced neurotoxicity [38, 64–71], our data rather support a model where Aβ-induced neurotoxicity is mainly executed by events occurring at the plasma membrane of neurons.

### Cytoplasmic N1-SP Does Not Behave Like a Cell-Penetrating Peptide

The shortest N-terminal PrP fragment shown to behave as a bona fide prion is caused by a stop mutation at aa145 and found in Gerstmann-Sträussler-Scheinker syndrome [126]. However, conversion and the infectious character of this PrP mutant depends on residues 112–139 which are lacking in our N1 construct. In fact, the disordered N1 alone is unlikely to undergo prion-like misfolding or mediate toxic effects. Yet there have been reports showing that cytPrP forms with uncleaved SP or artificially expressed PrP fragments composed of the SP and the first few residues of the charged cluster (PrP1–28 or PrP1–30) behave as CPPs [33–35]. Such PrP versions were shown to form intracellular aggregates, to destabilize the cytoskeleton and to exert membrane perturbations [35, 104, 127]. Fitting to the latter, when attached to the outer leaflet of the plasma membrane, the N-terminal part of PrP^C^ alone was shown to induce ionic currents, which might be associated with integration into membranes [29]. In strong contrast to these harmful effects, other studies have linked the PrP-derived CPPs with anti-prion properties as they have found reduced PrP^Sc^ levels in infected cells treated with these CPPs [128, 129].

Our transgenic mice showed cytosolic retention of—what we consider to be—N1-SP, which by containing the above-mentioned sequences would potentially qualify as a CPP. However, we did not find any evidence for detrimental effects such as an overtly disturbed cytoskeleton (as judged by the morphology of cerebral neurons in brain sections or primary neurons (e.g., MAP2)), dendritic spine loss or altered signaling that would recapitulate earlier findings made for PrP1–30 [35]. This indicates that cytosolic N1-SP is unable to penetrate membranes and to exert deleterious effects. As discussed above, we also did not find any protective anti-prion action of cytosolic N1-SP described earlier or exogenously administered PrP-CPPs [128, 129]. Fittingly, while N1 was shown to enter the cytosol when applied to cell culture supernatants (thus confirming its capacity to penetrate membranes), protective effects of N1 did not depend on this internalization but rather on its extracellular presence [61]. The latter study also showed involvement of the Akt pathway in the neuroprotection mediated by extracellular N1; yet, cytoplasmic N1 in our transgenic mice did not cause alterations in this cascade.

### Conclusions and Outlook

Our study provides the first in vivo proof of the impaired ER translocation of IDDs, in this case resulting in the aberrant retention of N1-SP in the cytoplasm. As such, this TgN1 animal model may become a valuable resource to study mechanisms and consequences of a hindered ER import despite presence of an ER-targeting SP and an active translocon. Moreover, this model may provide in vivo insight into the interaction between the PrP^C^ N-terminus and RNAs and its role in phase separation processes in the cytoplasm [130, 131].

Although cytosolic N1-SP contains the relevant CPP motif described by others, it neither causes overtly deleterious perturbations of intracellular membranes, the cytoskeleton or signaling pathways, nor does it exert any anti-prion effect previously ascribed to shorter PrP-derived CPPs. This supports the view that protective effects of N1 are strongly dependent on its presence in the extracellular space, i.e., its physiological localization endogenously ensured by the constitutive α-cleavage of PrP^C^. Since the identity of the responsible protease(s) is unclear, transgenic overexpression of N1 remains a valid strategy to study its protective effects against neurodegeneration in vivo. However, findings highlighted here, such as the need for structured C-terminal tags to allow for efficient translocation/secretion and to avoid degradative proteolytic trimming, or the extreme tolerance of the α-PrPase(s) to C-terminal modifications, have to be considered. As a consequence, we have recently generated transgenic mice overexpressing N1Fc that will likely provide further insight into N1-associated protection against neurodegenerative diseases and beyond.

## Electronic Supplementary Material

Suppl. Fig. 1 **a** Replica western blots of brain homogenates derived from PrP-KO (*Prnp*^0/0^), control (WT), TgN1 and PrP^C^-overexpressing (Tga20) mice detected with PrP-directed antibodies having epitopes in the N-terminal (POM2, 6D11) or C-terminal (POM1, EP1802Y) half of the protein (as introduced in Fig. 1). A dotted line was drawn for better comparison of fragment sizes. Both “N-terminal antibodies” detect a band of ~20 kDa that clearly correlates with PrP^C^ expression levels and is not detected with POM1 and EP1802Y, possibly representing the endogenous N3 fragment. POM1 and EP1802Y instead detect different glycoforms of the truncated C1 fragment with the unglycosylated C1 running at ~15 kDa (as shown in **c**). Notably, smaller fragments around 10 kDa, likely representing N1, are only detected with POM2 and 6D11. Note that TgN1 samples, as in Fig. 2e,f, present with a slightly higher band indicative of N1 with an uncleaved signal peptide (N1-SP). A weak band (observed with POM2 in Tga20; indicated by #) slightly lower than N1 might reflect the endogenous N2 fragment (β-cleavage) or result from sequential “N1 trimming” (see Suppl. Fig. 4a). An asterisk indicates a presumably unspecific band observed in PrP-KO when detected with POM2. **b** Size comparison of N-terminal fragments detected in WT and TgN1 brain homogenates (POM2 antibody was used here) with recombinant (human) N1. Despite only weak detection of N-terminal fragments in these brain samples, presence of a band running slightly higher than N1 further supports presence of N1-SP in TgN1 mice. Again, the # marks a smaller band (N2 or “trimmed N1”) detected upon longer exposure. **c** Assessment of levels of the endogenous C1 fragment (~15 kDa) resulting from α-cleavage upon deglycosylation (PNGase F digestion). POM1 antibody was used for detection. No differences were observed between WT controls (set to 1) and TgN1 mice (*p*=0.9; n=3) (PNG 1459 kb)

High Resolution Image (TIF 1482 kb)

Suppl. Fig. 2 No alterations in candidate PrP-associated signaling pathways between TgN1 and WT mice. Forebrain homogenates of 43 weeks old mice were analyzed for the phosphorylation state of certain signaling pathways. No significant changes between genotypes were observed for Akt, eIF2α, Erk1/2 and p38. Densitometric quantification (on the right) shows the direct ratio of phosphorylated (P) versus total levels when both forms were assessed on the same blot (Akt), whereas ratios were made only after normalization to corresponding actin signals when run on parallel blots. “M” indicates the middle position of a size marker lane in some of the blots. Note that, in some occasions, the molecular weight marker bands caused an unspecific signal in the red channel (PNG 1844 kb)

High Resolution Image (TIF 2221 kb)

Suppl. Fig. 3 Immunohistochemical assessment of spongiosis and glial activation in prion-diseased versus control mice. Astrocytes (GFAP) and microglia (Iba1) are highly upregulated in terminally prion diseased (+RML) TgN1 and WT control mice compared to age-matched controls (+CD1) without prion infection. Likewise, spongiform lesions (vacuoles detected in the H&E stained sections) are only observed in prion-infected mice. Hippocampal and cerebellar brain regions are shown for representation. No overt differences were observed between genotypes (n=4). Scale bar represents 100 μm. Note that tissue disruption in some samples is due to technical issues but does not interfere with the overall scientific assessment (PNG 1495 kb)

High Resolution Image (TIF 3115 kb)

Suppl. Fig. 4 **a** Low biostability and fast degradation of N1. Western blot analysis and schematic representation of WT N2a cells and conditioned media treated either with an antibody directed against the C-terminus of N1 generated upon α-cleavage (6D11; epitope ranging from amino acid 93 to 109) or with an antibody (POM1) against the globular C-terminal part (sAPPα was detected as loading control for media samples). N1 is recovered at the expected size (~10 kDa) when “protected” by binding of 6D11, whereas a fragmentation to lower molecular weight bands is observed with the control treatment (POM1). This indicates a proteolytic trimming of N1 at its C-terminus once it is released from cells (scheme on the right). **b** Western blot analysis of lysates and respective media supernatants of PrP-depleted N2a cells upon transfection (TF) with constructs coding for PrP, N1, or N1 fused to an IgG Fc part (N1Fc) or a nanobody (N1Nb). In contrast to N1 alone (which is mostly found inside the cells), N1Fc and N1Nb are efficiently secreted into the media. Note that besides the full-length forms of both fusion proteins, there is also a clear band for the N1 fragment in these lanes indicating that an α-cleavage-like event also occurs on these fusion proteins. Detection was with the 6D11 antibody. Lower blot: Besides confirming these findings, a replica experiment, yet detected with the POM2 antibody instead, more clearly reveals the presence of a band running higher than N1 in lysates of N1-transfected cells, thus supporting the presence of cytosolic N1-SP. The asterisk indicates a band of unknown identity (PNG 1610 kb)

High Resolution Image (TIF 2192 kb)

ESM ESM 1 (DOCX 21.5 kb)

## Abbreviations

Aβ: amyloid-beta peptide
AD: Alzheimer’s disease
CPP: cell-penetrating peptide
GPI: glycosylphosphatidylinositol (anchor)
IDD: intrinsically disordered domain
IDP: intrinsically disordered protein/peptide
N1: prion protein N-terminal fragment
PD: Parkinson’s disease
PrP^C^: cellular prion protein
PrP^Sc^: pathogenic isoform of the prion protein
SP: signal peptide
UPS: ubiquitin-proteasome system
