# Supplementary figures and images for: Transgenic Overexpression of the Disordered Prion Protein N1 Fragment in Mice Does Not Protect Against Neurodegenerative Diseases Due to Impaired ER Translocation
Source: Mol Neurobiol. 2020 May 4;57(6):2812–29. doi: 10.1007/s12035-020-01917-2 (PMC7253391; doi:10.1007/s12035-020-01917-2)

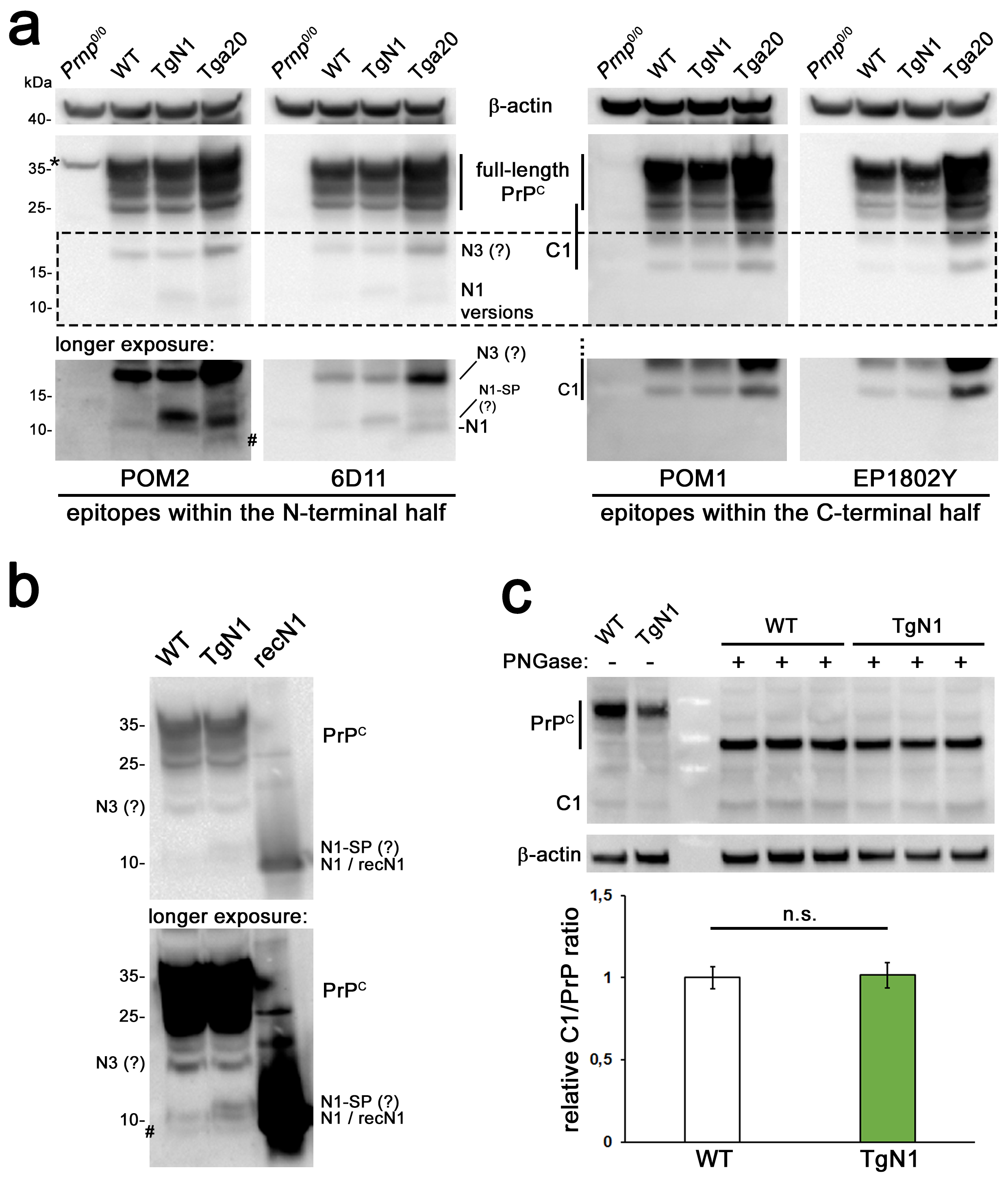

Supplement: Supplementary file 1 — a Replica western blots of brain homogenates derived from PrP-KO (Prnp0/0), control (WT), TgN1 and PrPC-overexpressing (Tga20) mice detected with PrP-directed antibodies having epitopes in the N-terminal (POM2, 6D11) or C-terminal (POM1, EP1802Y) half of the protein (as introduced in Fig. 1). A dotted line was drawn for better comparison of fragment sizes. Both “N-terminal antibodies” detect a band of ~20 kDa that clearly correlates with PrPC expression levels and is not detected with POM1 and EP1802Y, possibly representing the endogenous N3 fragment. POM1 and EP1802Y instead detect different glycoforms of the truncated C1 fragment with the unglycosylated C1 running at ~15 kDa (as shown in c). Notably, smaller fragments around 10 kDa, likely representing N1, are only detected with POM2 and 6D11. Note that TgN1 samples, as in Fig. 2e,f, present with a slightly higher band indicative of N1 with an uncleaved signal peptide (N1-SP). A weak band (observed with POM2 in Tga20; indicated by #) slightly lower than N1 might reflect the endogenous N2 fragment (β-cleavage) or result from sequential “N1 trimming” (see Suppl. Fig. 4a). An asterisk indicates a presumably unspecific band observed in PrP-KO when detected with POM2. b Size comparison of N-terminal fragments detected in WT and TgN1 brain homogenates (POM2 antibody was used here) with recombinant (human) N1. Despite only weak detection of N-terminal fragments in these brain samples, presence of a band running slightly higher than N1 further supports presence of N1-SP in TgN1 mice. Again, the # marks a smaller band (N2 or “trimmed N1”) detected upon longer exposure. c Assessment of levels of the endogenous C1 fragment (~15 kDa) resulting from α-cleavage upon deglycosylation (PNGase F digestion). POM1 antibody was used for detection. No differences were observed between WT controls (set to 1) and TgN1 mice (p=0.9; n=3) (PNG 1459 kb) [file 12035_2020_1917_Fig8_ESM.png]

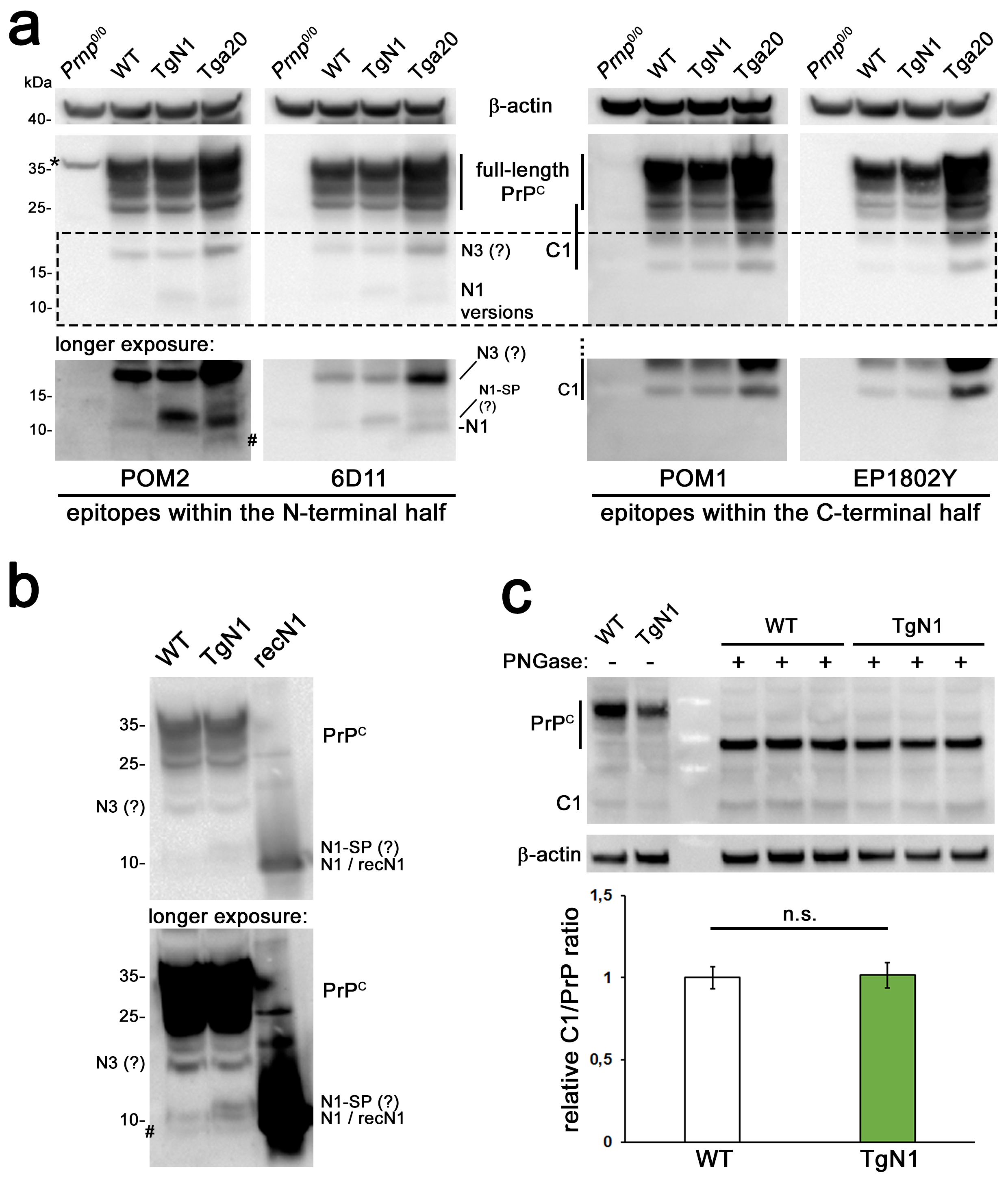

Supplement: Supplementary file 2 — High Resolution Image (TIF 1482 kb) [file 12035_2020_1917_MOESM1_ESM.tif]

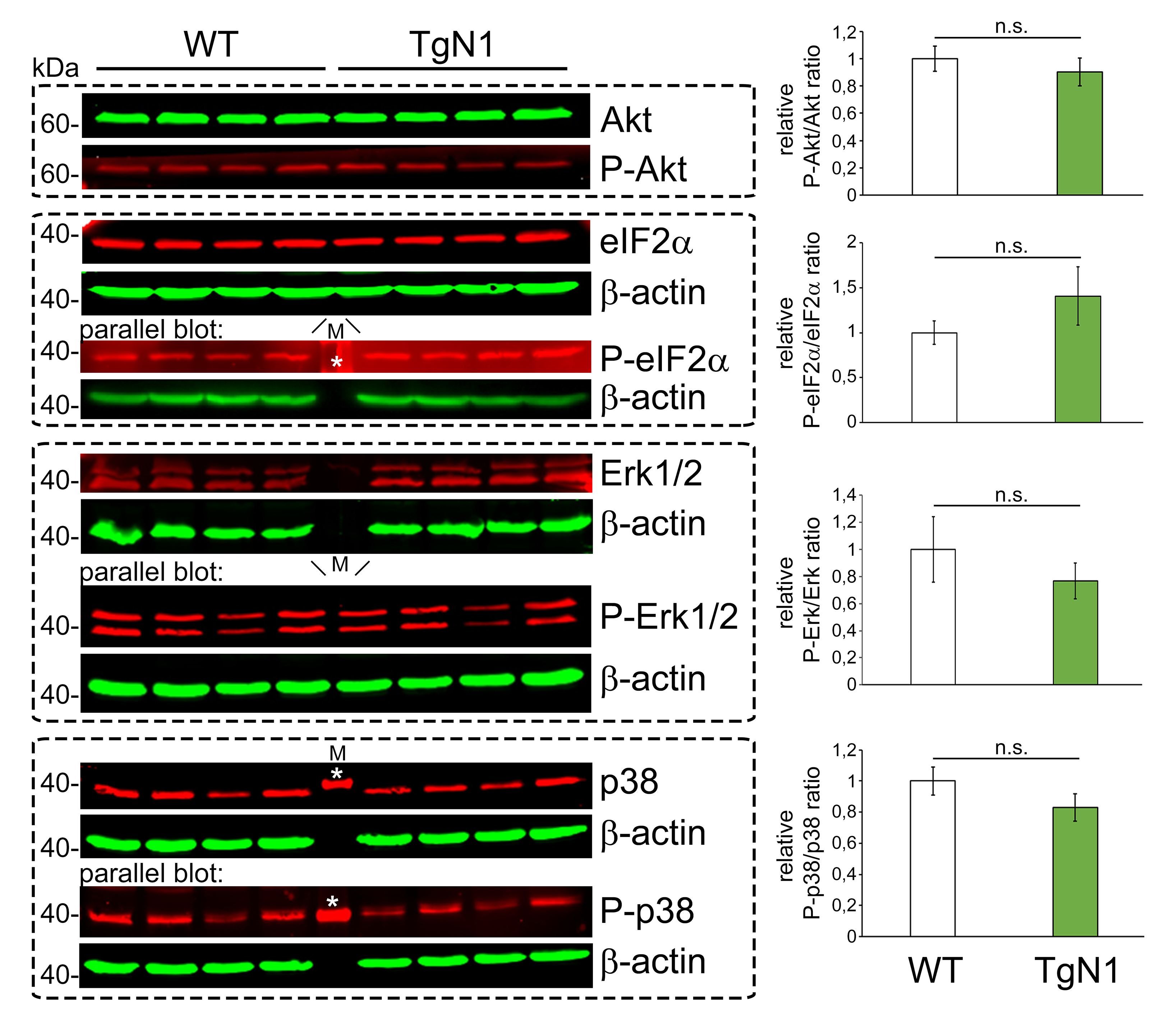

Supplement: Supplementary file 3 — No alterations in candidate PrP-associated signaling pathways between TgN1 and WT mice. Forebrain homogenates of 43 weeks old mice were analyzed for the phosphorylation state of certain signaling pathways. No significant changes between genotypes were observed for Akt, eIF2α, Erk1/2 and p38. Densitometric quantification (on the right) shows the direct ratio of phosphorylated (P) versus total levels when both forms were assessed on the same blot (Akt), whereas ratios were made only after normalization to corresponding actin signals when run on parallel blots. “M” indicates the middle position of a size marker lane in some of the blots. Note that, in some occasions, the molecular weight marker bands caused an unspecific signal in the red channel (PNG 1844 kb) [file 12035_2020_1917_Fig9_ESM.png]

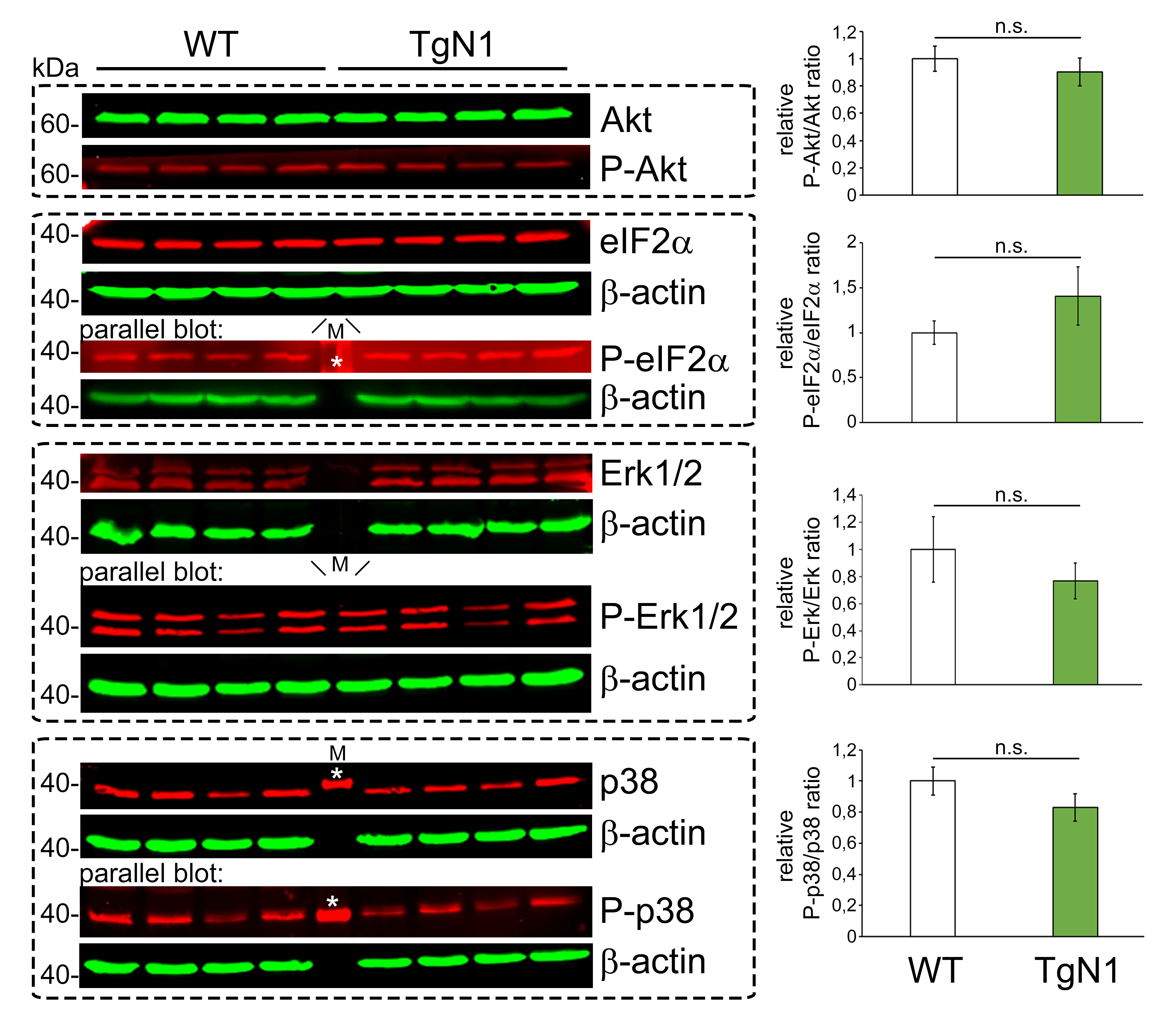

Supplement: Supplementary file 4 — High Resolution Image (TIF 2221 kb) [file 12035_2020_1917_MOESM2_ESM.tif]

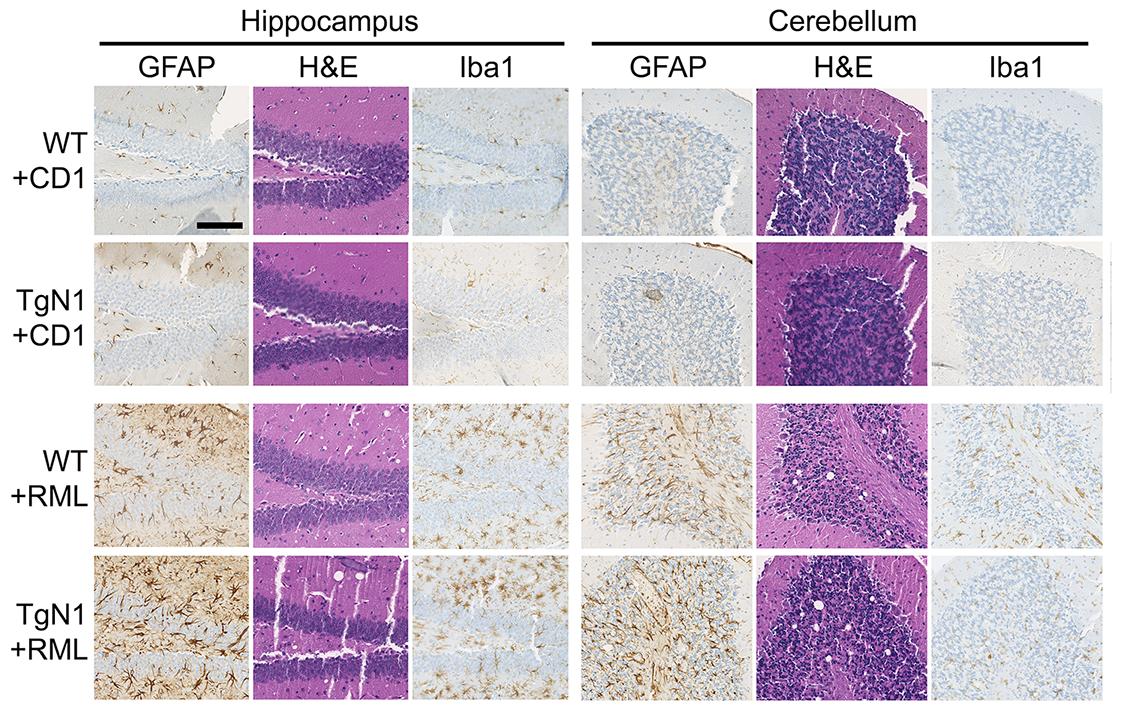

Supplement: Supplementary file 5 — Immunohistochemical assessment of spongiosis and glial activation in prion-diseased versus control mice. Astrocytes (GFAP) and microglia (Iba1) are highly upregulated in terminally prion diseased (+RML) TgN1 and WT control mice compared to age-matched controls (+CD1) without prion infection. Likewise, spongiform lesions (vacuoles detected in the H&E stained sections) are only observed in prion-infected mice. Hippocampal and cerebellar brain regions are shown for representation. No overt differences were observed between genotypes (n=4). Scale bar represents 100 μm. Note that tissue disruption in some samples is due to technical issues but does not interfere with the overall scientific assessment (PNG 1495 kb) [file 12035_2020_1917_Fig10_ESM.png]

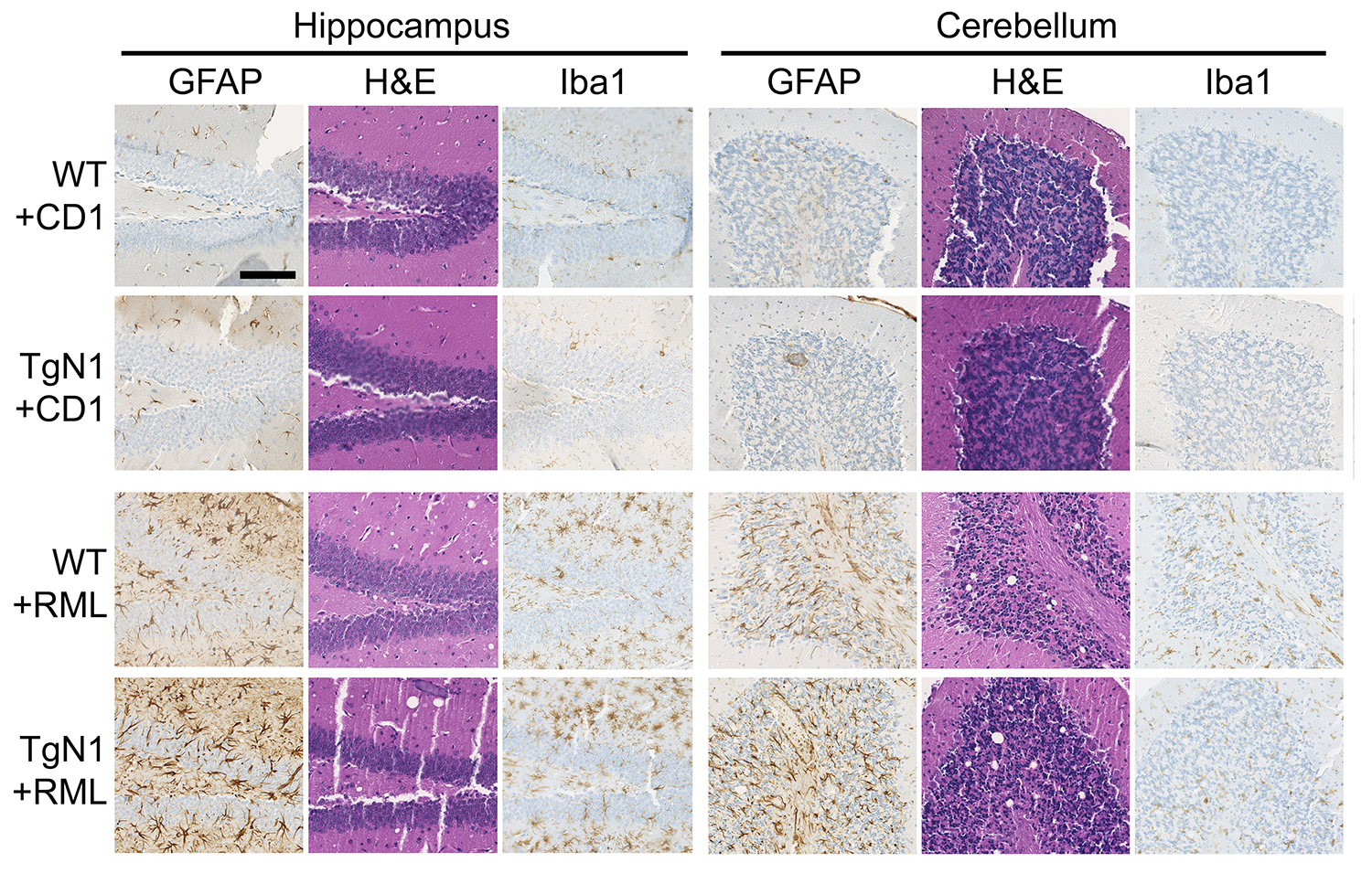

Supplement: Supplementary file 6 — High Resolution Image (TIF 3115 kb) [file 12035_2020_1917_MOESM3_ESM.tif]

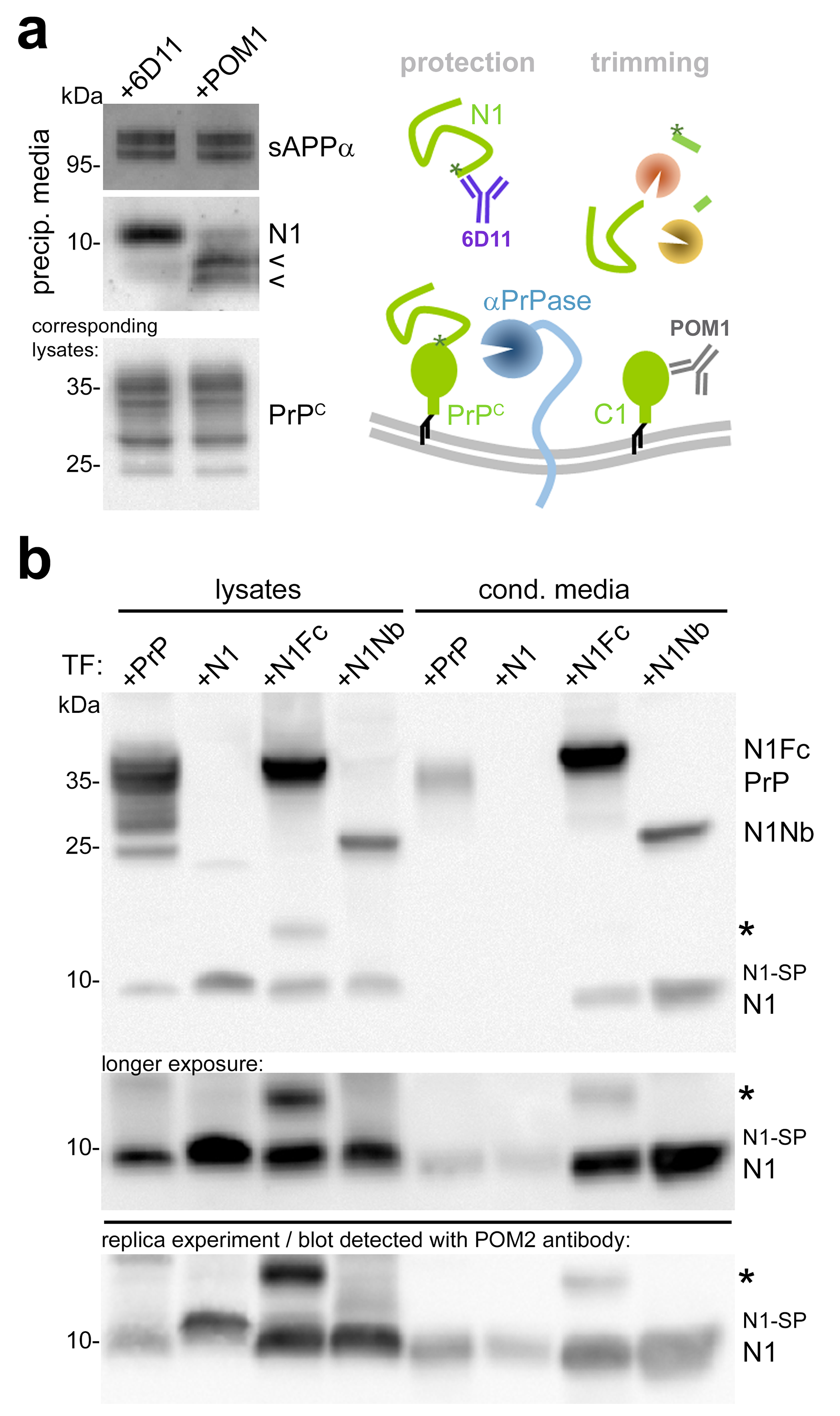

Supplement: Supplementary file 7 — a Low biostability and fast degradation of N1. Western blot analysis and schematic representation of WT N2a cells and conditioned media treated either with an antibody directed against the C-terminus of N1 generated upon α-cleavage (6D11; epitope ranging from amino acid 93 to 109) or with an antibody (POM1) against the globular C-terminal part (sAPPα was detected as loading control for media samples). N1 is recovered at the expected size (~10 kDa) when “protected” by binding of 6D11, whereas a fragmentation to lower molecular weight bands is observed with the control treatment (POM1). This indicates a proteolytic trimming of N1 at its C-terminus once it is released from cells (scheme on the right). b Western blot analysis of lysates and respective media supernatants of PrP-depleted N2a cells upon transfection (TF) with constructs coding for PrP, N1, or N1 fused to an IgG Fc part (N1Fc) or a nanobody (N1Nb). In contrast to N1 alone (which is mostly found inside the cells), N1Fc and N1Nb are efficiently secreted into the media. Note that besides the full-length forms of both fusion proteins, there is also a clear band for the N1 fragment in these lanes indicating that an α-cleavage-like event also occurs on these fusion proteins. Detection was with the 6D11 antibody. Lower blot: Besides confirming these findings, a replica experiment, yet detected with the POM2 antibody instead, more clearly reveals the presence of a band running higher than N1 in lysates of N1-transfected cells, thus supporting the presence of cytosolic N1-SP. The asterisk indicates a band of unknown identity (PNG 1610 kb) [file 12035_2020_1917_Fig11_ESM.png]

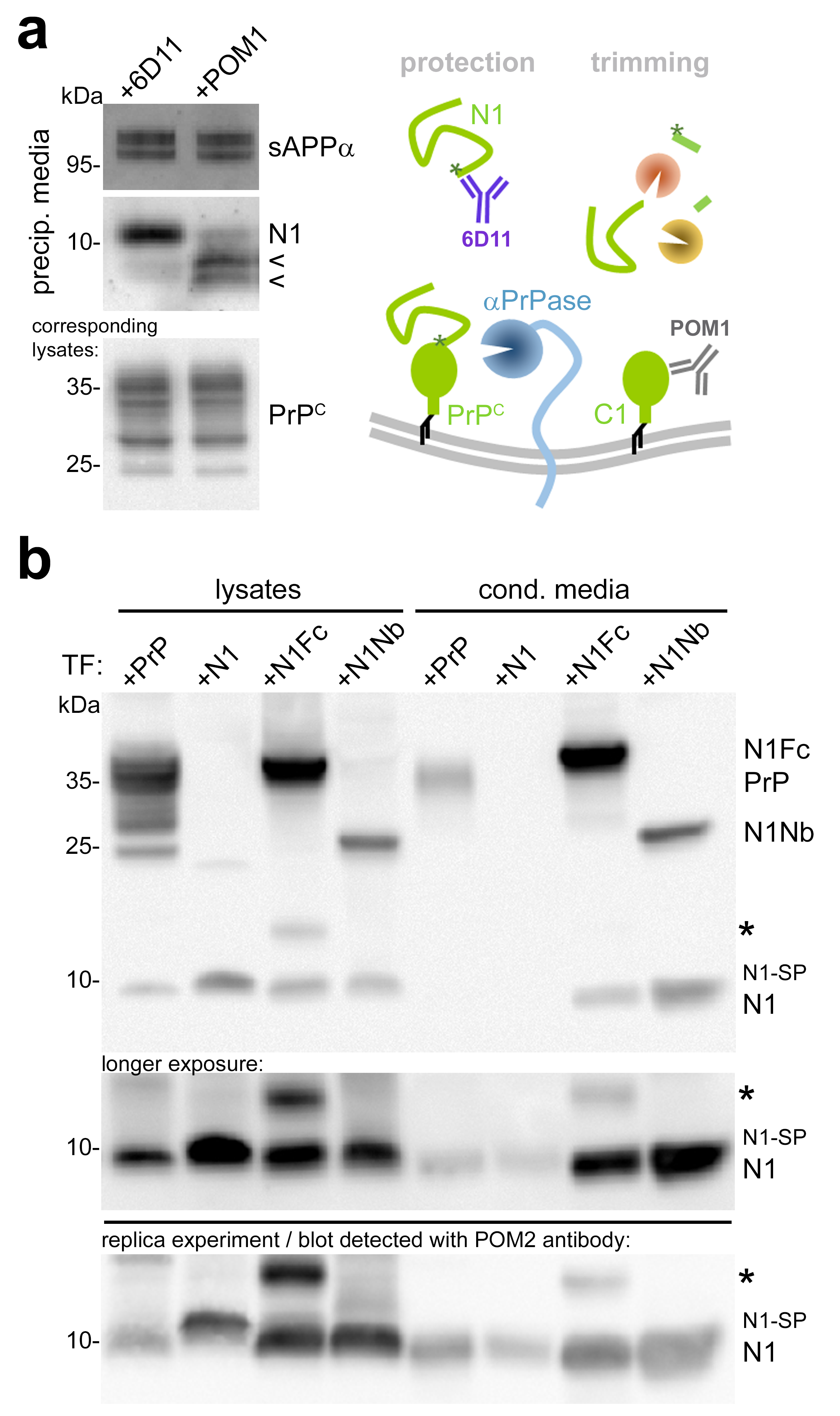

Supplement: Supplementary file 8 — High Resolution Image (TIF 2192 kb) [file 12035_2020_1917_MOESM4_ESM.tif]
