# Supplementary material for: Transgenic Overexpression of the Disordered Prion Protein N1 Fragment in Mice Does Not Protect Against Neurodegenerative Diseases Due to Impaired ER Translocation
Source: Mol Neurobiol. 2020 May 4;57(6):2812–29. doi: 10.1007/s12035-020-01917-2 (PMC7253391; doi:10.1007/s12035-020-01917-2)
Supplement: Supplementary file 9 — (DOCX 21.5 kb) [file 12035_2020_1917_MOESM5_ESM.docx]

**Supplementary table 1**

**(a) List of antibodies**

| **Name** | **Cat.#** | **Company** | **Species** | **Dilution** | **Blocking and buffer** |
| --- | --- | --- | --- | --- | --- |
| ***Antibodies for immunoblots*** |  |  |  |  |  |
| **p44/42 MAPK (Erk1/2) (137F5)** | 4695 | Cell Signaling | Rabbit | 1:1000 | 5% BSA in TBST |
| **P-p44/42 MAPK (Erk1/2) (Thr202/Tyr204)** | 9101 | Cell Signaling | Rabbit | 1:1000 | 5% BSA in TBST |
| **p38 MAPK Antibody** | 9212 | Cell Signaling | Rabbit | 1:1000 | 5% BSA in TBST |
| **P-p38 MAPK (Thr180/Tyr182) (D3F9)** | 4511 | Cell Signaling | Rabbit | 1:1000 | 5% BSA in TBST |
| **eIF2a Antibody** | 9722 | Cell Signaling | Rabbit | 1:1000 | 5% BSA in TBST |
| **P-eIF2a (Ser51) Antibody** | 9721 | Cell Signaling | Rabbit | 1:1000 | 5% BSA in TBST |
| **Fyn Antibody** | 4023 | Cell Signaling | Rabbit | 1:1000 | 5% BSA in TBST |
| **P-Src Family (Tyr416) Antibody** | 2101 | Cell Signaling | Rabbit | 1:1000 | 5% BSA in TBST |
| **Akt (pan) (40D4) Mouse mAb** | 2920 | Cell Signaling | Mouse | 1:2000 | 5% milk in TBST |
| **P-Akt (Ser473) Antibody** | 9271 | Cell Signaling | Rabbit | 1:1000 | 5% BSA in TBST |
| **POM1 (anti-PrP)** | - | Prof. Dr. Aguzzi, Zürich, Switzerland | Mouse | 1:2000 | in 1x Roti block |
| **POM2 (anti-PrP)** | - | Prof. Dr. Aguzzi, Zürich, Switzerland | Mouse | 1:2000 | in 1x Roti block |
| **6D11 (anti-PrP)** | 808001 | Biolegend | Mouse | 1:2000 | in 1x Roti block |
| **EP1802Y (anti-PrP)** | ab238428 | abcam | Rabbit | 1:1000 | in 1x Roti block |
| **anti mouse/rat beta amyloid (sAPP alpha)** | 826801 | Biolegend | Rabbit | 1:1000 | in 1x Roti block |
| **beta-actin(C4)** | MAB1501 | Millipore | Mouse | 1:3000 | in 1x Roti block |
| **beta-catenin (6B3)** | 9582s | Cell Signaling | Rabbit | 1:1000 | in 1x Roti block |
|  |  |  |  |  |  |
| ***Antibodies for IF stainings*** |  |  |  |  |  |
| **Synaptophysin** | ab32594 | abcam | Rabbit | 1:200 | 1% BSA in PBST |
| **Anti-MAP2 antibody** | M9942 | Sigma-Aldrich | Mouse | 1:500 | 1% BSA in PBST |
| **Golgi marker (GM130)** | ab52649 | abcam | Rabbit | 1:100 | 1% FBS / 0.1% glycine / 0.1% saponin in PBS |
| **POM1** |  | Prof. Dr. Aguzzi, Zürich, Switzerland | Mouse | 1:200 | 1% FBS / 0.1% glycine / 0.1% saponin in PBS |
| **POM2** |  | Prof. Dr. Aguzzi, Zürich, Switzerland | Mouse | 1:200 | 1% FBS / 0.1% glycine / 0.1% saponin in PBS |
|  |  |  |  |  |  |
| ***Secondary antibodies*** |  |  |  |  |  |
| **IRDye 680 RD Donkey anti-Rabbit IgG** | 925-68073 | LICOR | Donkey | 1:10000 | in 1x Roti block |
| **IRDye 800 CW Donkey anti-mouse IgG** | 925-32212 | LICOR | Donkey | 1:10000 | in 1x Roti block |
| **Anti rabbit IgG HRP conjugate** | W401B | Promega | anti Rabbit | 1:5000 | in 1x Roti block |
| **Anti mouse IgG HRP conjugate** | W402B | Promega | anti Mouse | 1:5000 | in 1x Roti block |
| **Donkey anti-Rabbit IgG (H+L) Secondary Antibody,**  **Alexa Fluor 488** | R37118 | Invitrogen | Donkey | 1:500 | 1% BSA in PBST |
| **Goat anti-Mouse IgG1 Cross-Adsorbed Secondary Antibody, Alexa Fluor 555** | A-21127 | Invitrogen | Goat | 1:500 | 1% BSA in PBST |

**(b) List of primers**

| **Name** | **Primer sequence** | **application** |
| --- | --- | --- |
| F N1 STOP | CAACCTCAAGCAT**TAG**GCAGGGGCTGCG | Mutagenesis at the α-cleavage site |
| R N1 STOP | CGCAGCCCCTGC**CTA**ATGCTTGAGGTTG | Mutagenesis at the α-cleavage site |
| F1-Exone2 | GAGCTGAAGCATTCTGCCTTCC | Forward primer for genotyping |
| R3-PrP | GATCTTCTCCCGTCGTAATAGGCCT | Reverse primer for genotyping |
| F-control PrP | ATGGCGAACCTTGGCTACTGGCT | Control Forward primer for genotyping |
| R-control PrP | CATCCCACGATCAGGAAGATGAGG | Control reverse primer for genotyping |
| R-HGC-Seq | TGTACATTTCCCAGGGCCCATCAGTGC | Reverse primer for sequencing the insert in the HGC |
| F-XhoI-N1 | GCTCTCTGGCTAACTCGAGAACCCACTGCTTACTG | Primers for taking out N1 cDNA from PrP in pcDNA3.1 |
| R-XhoI-N1 | CAGTAAGCAGTGGGTTCTCGAGTTAGCCAGAGAGC | Primers for taking out N1 cDNA from PrP in pcDNA3.1 |
| F-BglII-N1 | CTCAAGCATGTGGCAAGATCTGCGGCAGCTGGGG | Primers for taking out N1 cDNA from PrP in pcDNA3.1 |
| R--BglII-N1 | CCCCAGCTGCCGCAGATCTTGCCACATGCTTGAG | Primers for taking out N1 cDNA from PrP in pcDNA3.1 |
| F-mRPL13 | CGGAATGGCATGATACTGAAGCC | qPCR |
| R-mRPL13 | TTGGTGTGGTATCTCACTGTAGG | qPCR |
| F-N terminal | ATGGCGAACCTTGGCTACTG | qPCR |
| R-N terminal | CTGAGGTGGGTAACGGTTGC | qPCR |
